# Supplementary figures and images for: Selection in the dopamine receptor 2 gene: a candidate SNP study
Source: PeerJ. 2015 Aug 11;3:e1149. doi: 10.7717/peerj.1149 (PMC4540012; doi:10.7717/peerj.1149)

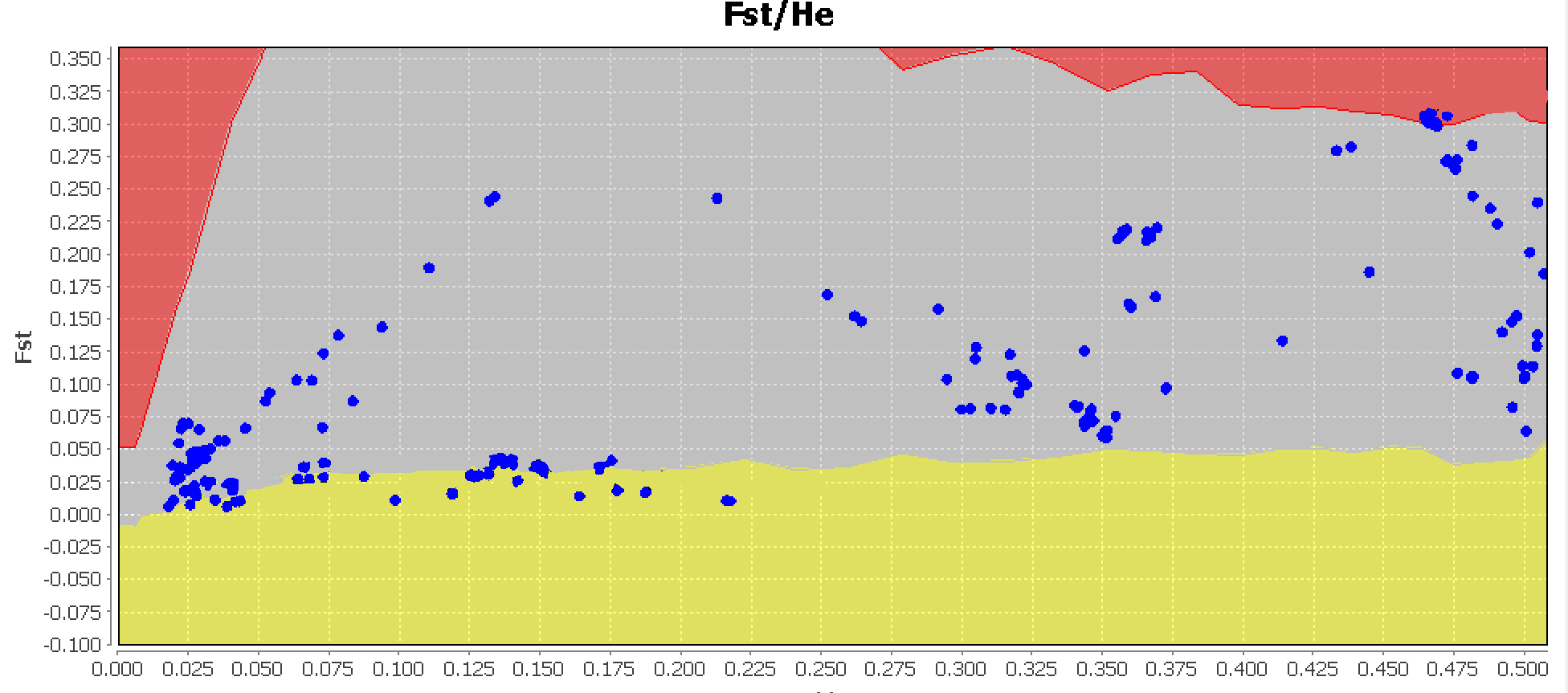

Supplement: Figure S1 — X-axis: estimated heterozygosity values. Y-axis: FST-values. The red area indicates positive directional selection, the grey area indicates neutrality, and the yellow area indicates balancing selection. Confidence intervals represent borders between “selection areas”. [file peerj-03-1149-s001.jpg]

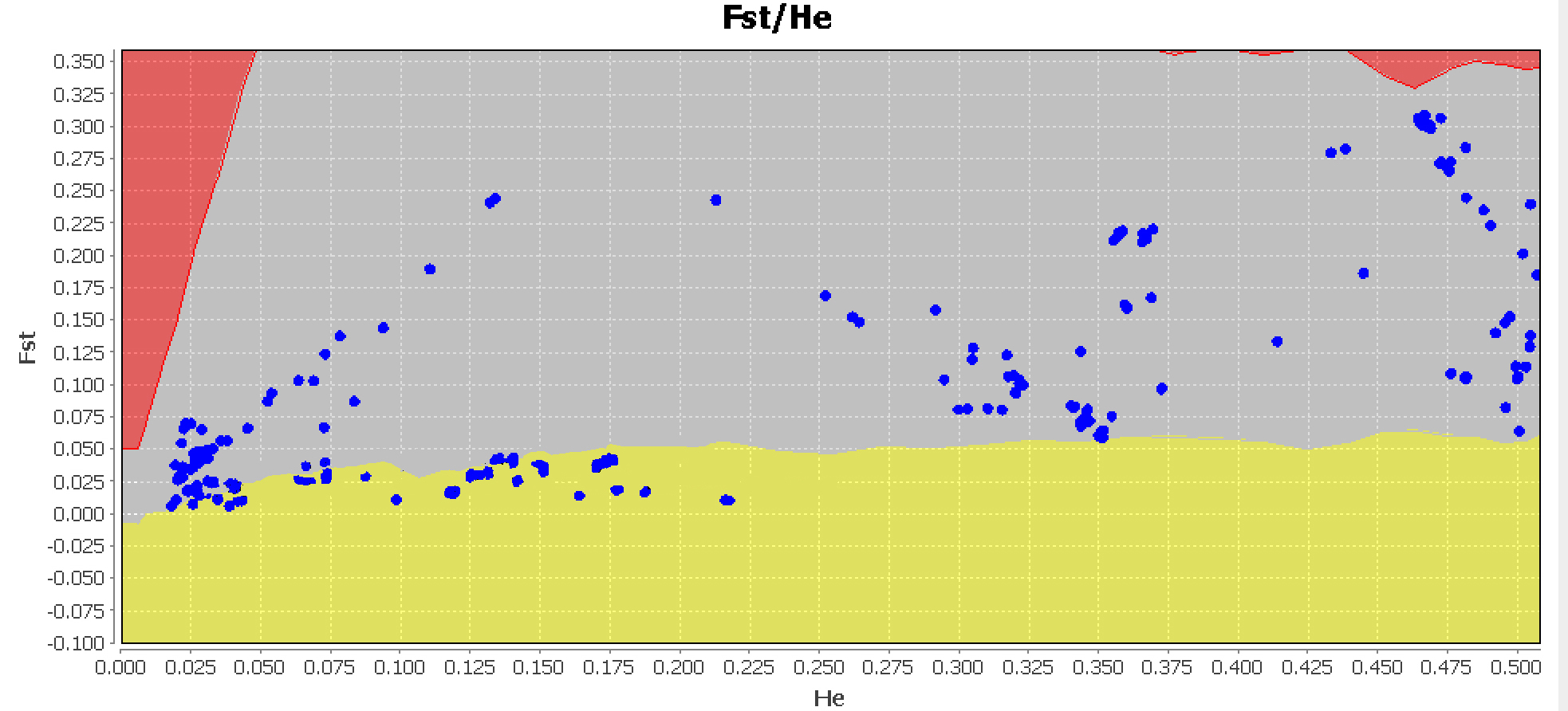

Supplement: Figure S2 — X-axis: estimated heterozygosity values. Y-axis: FST-values. The red area indicates positive directional selection, the grey area indicates neutrality, and the yellow area indicates balancing selection. Confidence intervals represent borders between “selection areas”. [file peerj-03-1149-s002.jpg]

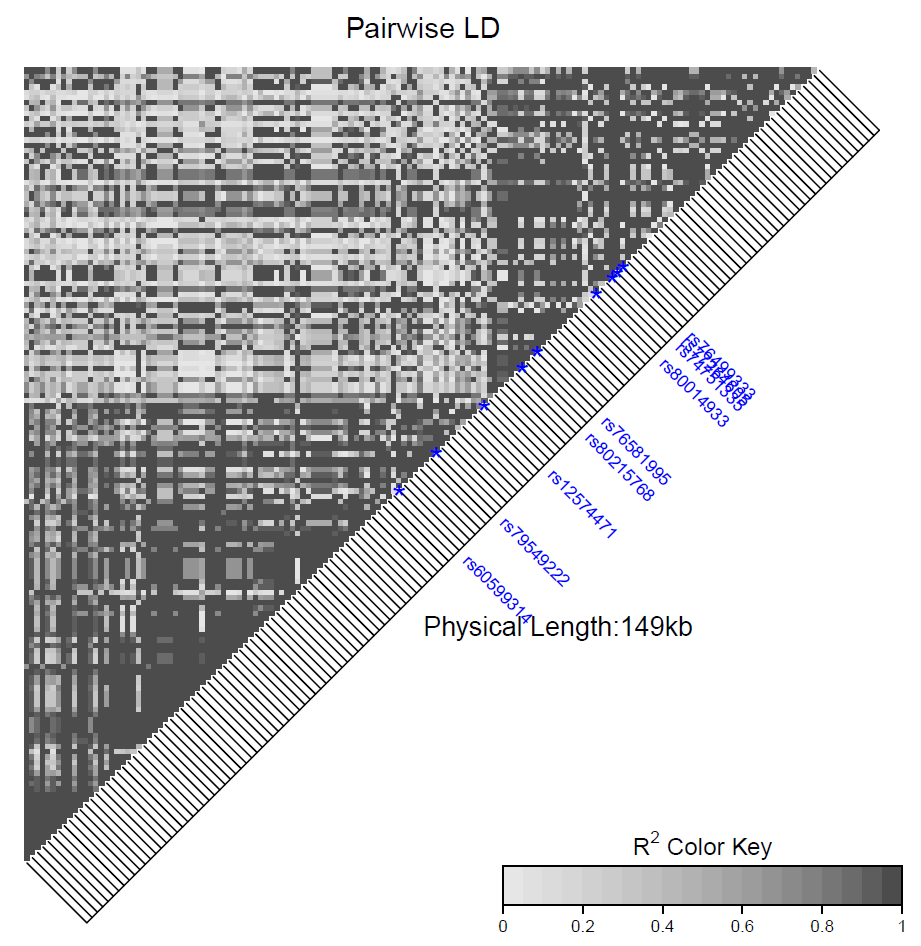

Supplement: Figure S3 [file peerj-03-1149-s003.png]

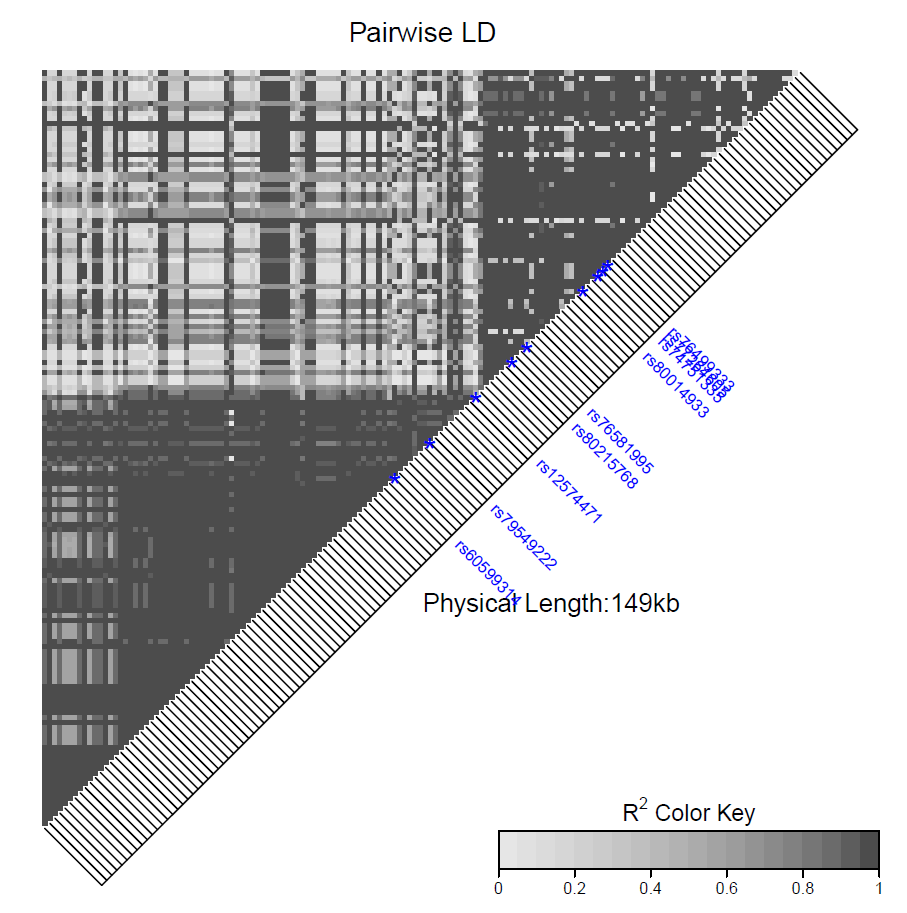

Supplement: Figure S4 [file peerj-03-1149-s004.png]

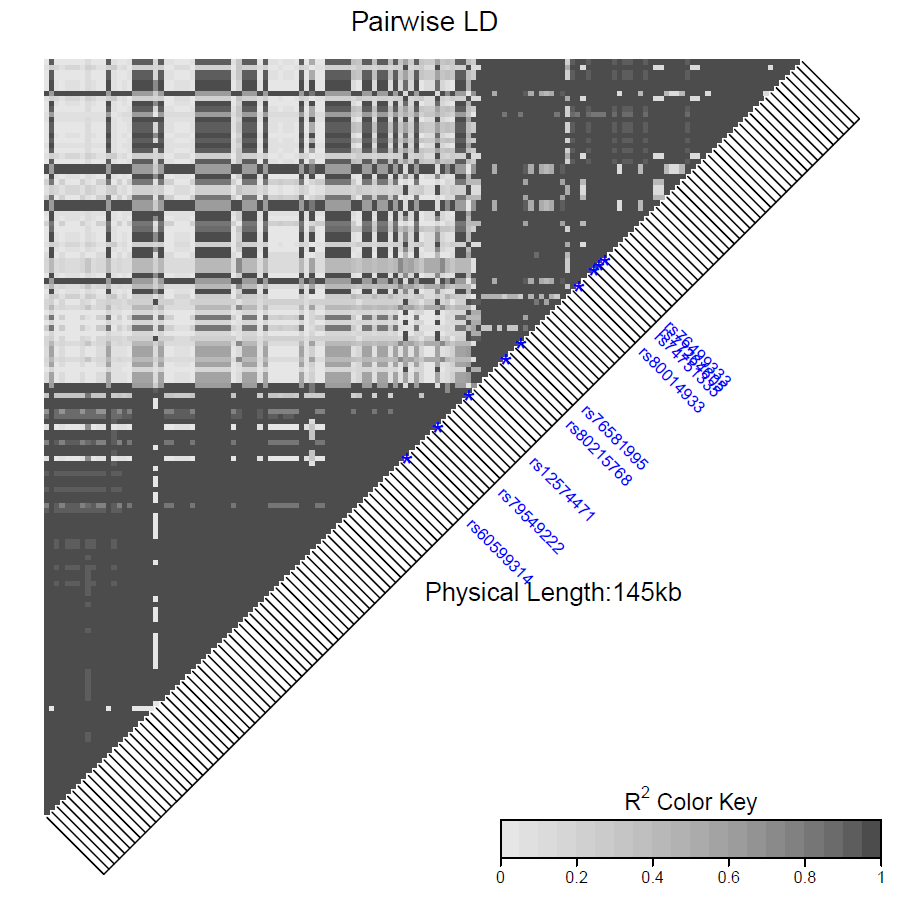

Supplement: Figure S5 [file peerj-03-1149-s005.png]

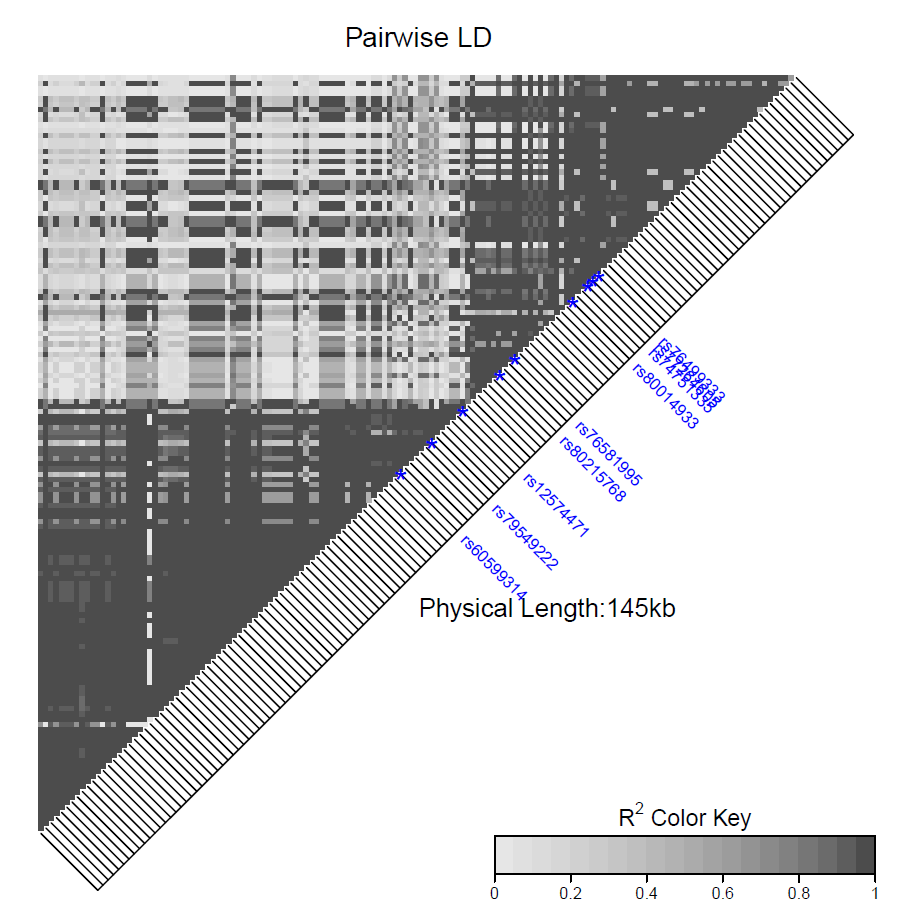

Supplement: Figure S6 [file peerj-03-1149-s006.png]

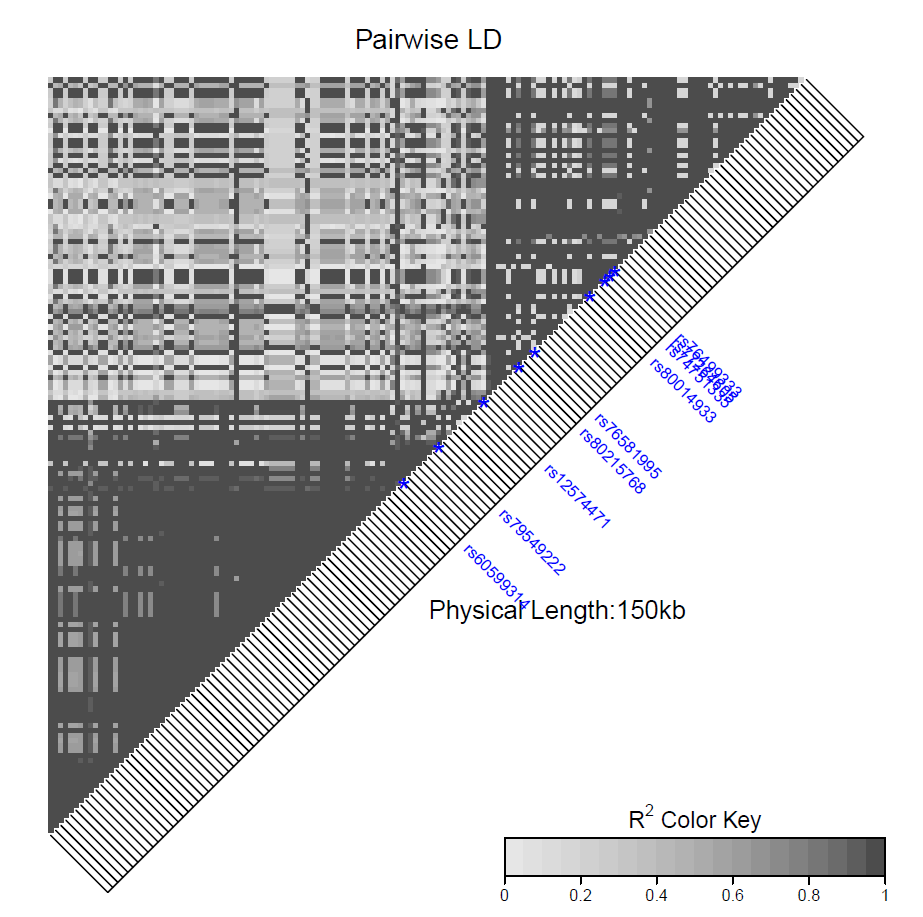

Supplement: Figure S7 [file peerj-03-1149-s007.png]

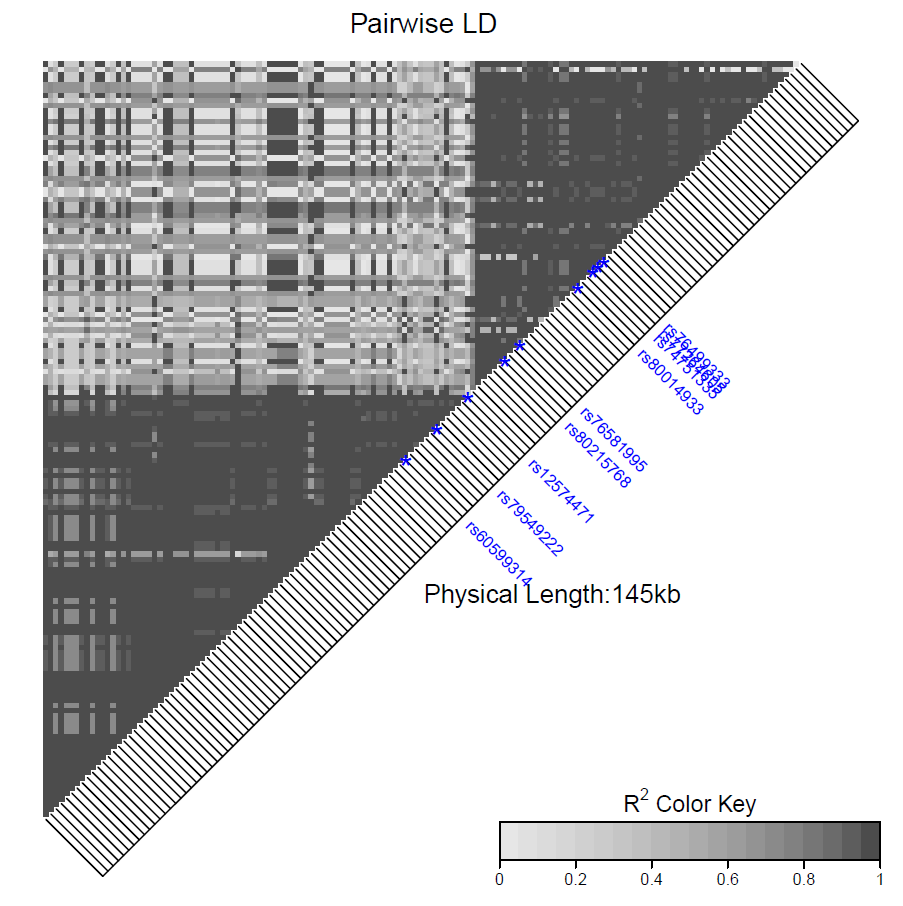

Supplement: Figure S8 [file peerj-03-1149-s008.png]

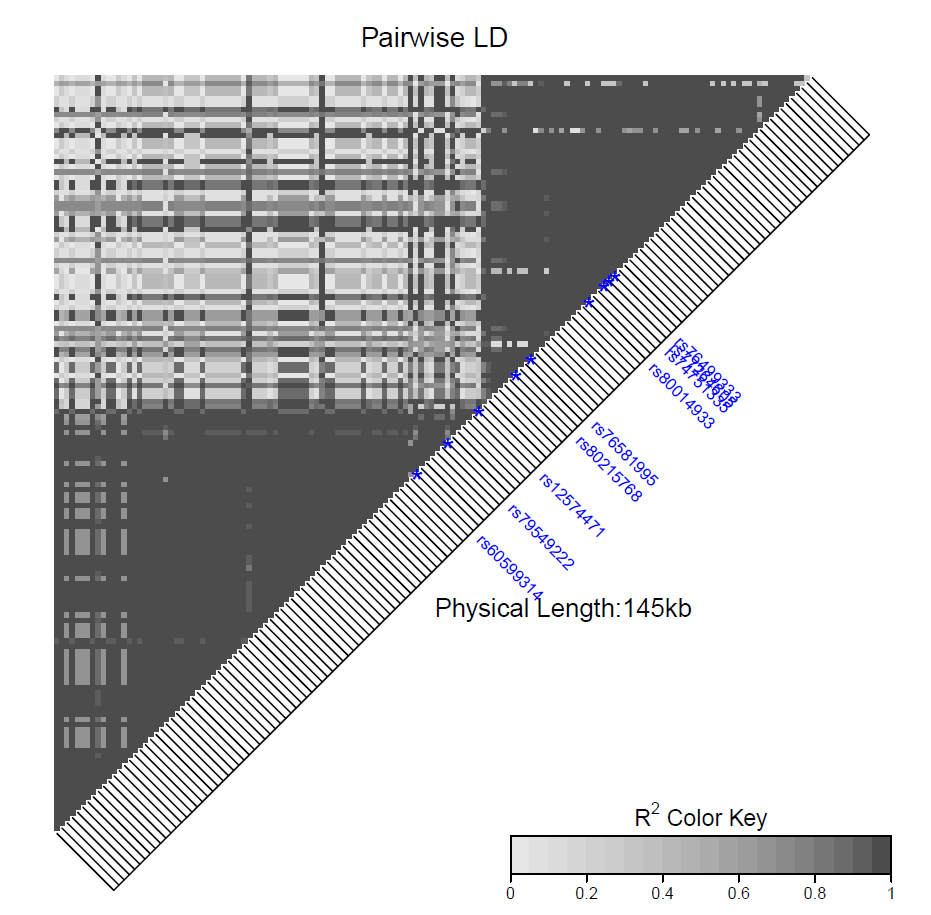

Supplement: Figure S9 [file peerj-03-1149-s009.png]

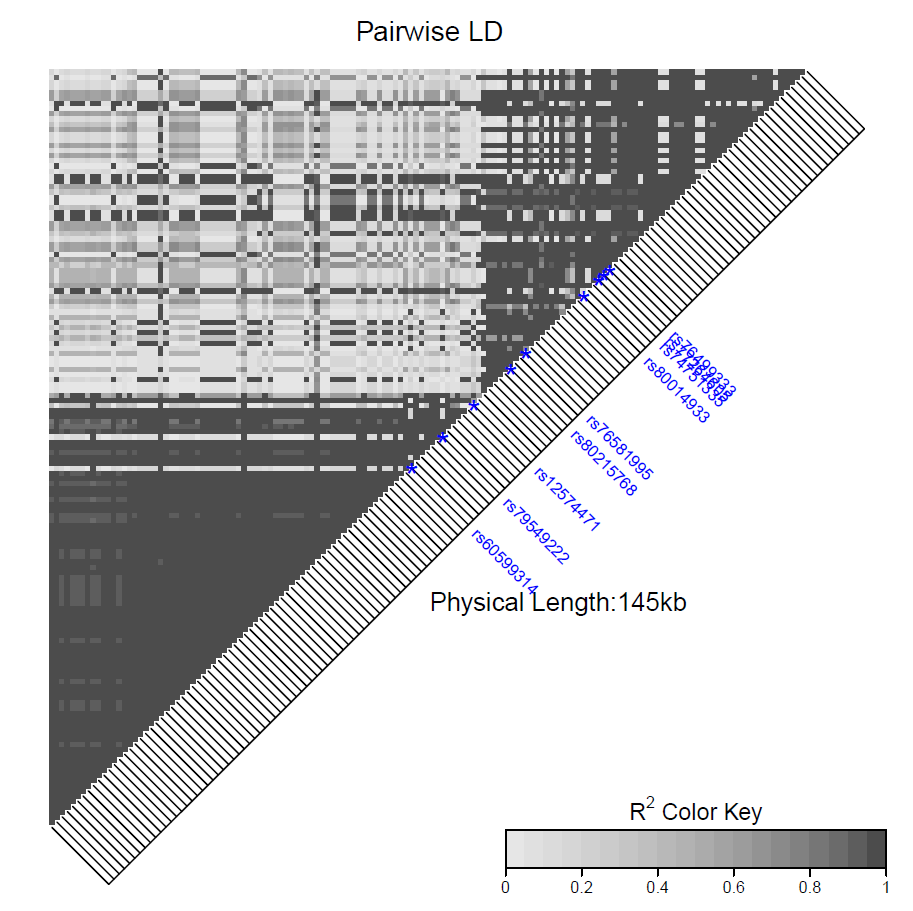

Supplement: Figure S10 [file peerj-03-1149-s010.png]

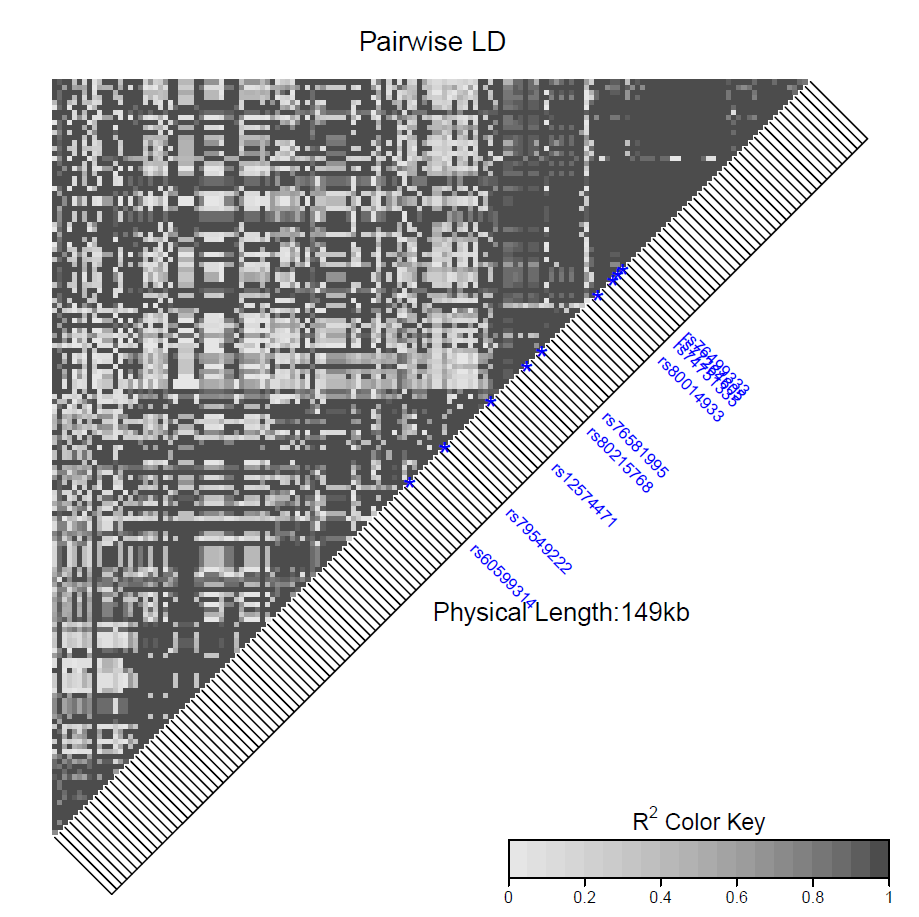

Supplement: Figure S11 [file peerj-03-1149-s011.png]

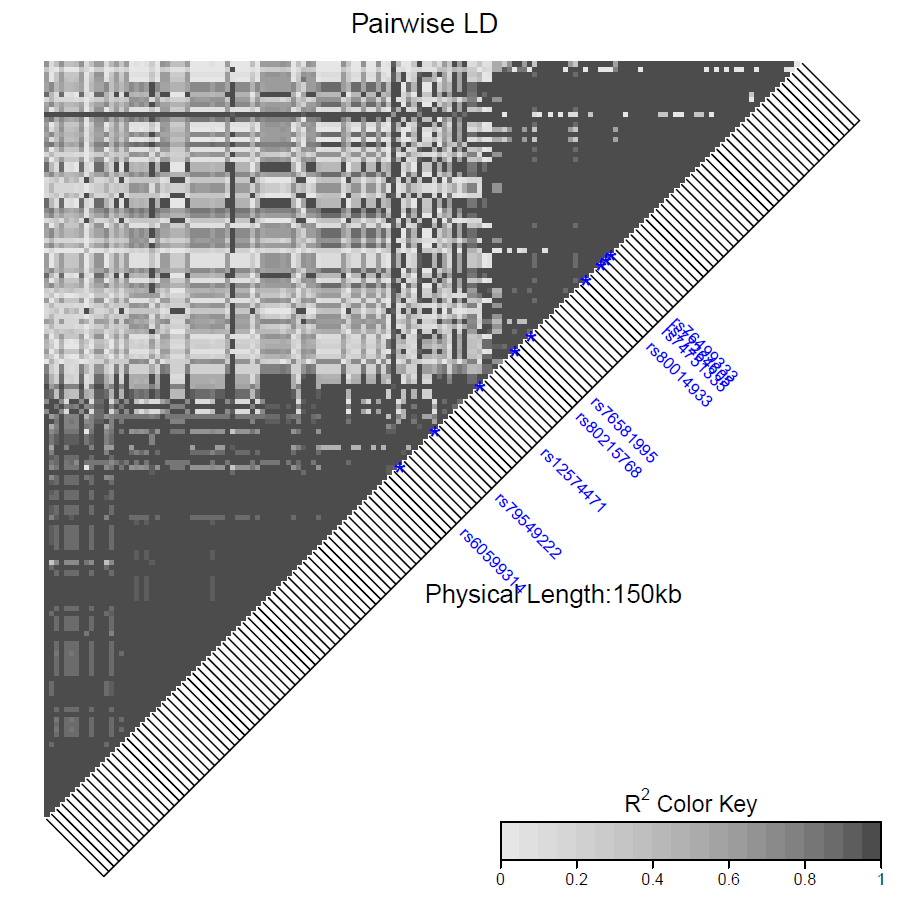

Supplement: Figure S12 [file peerj-03-1149-s012.png]

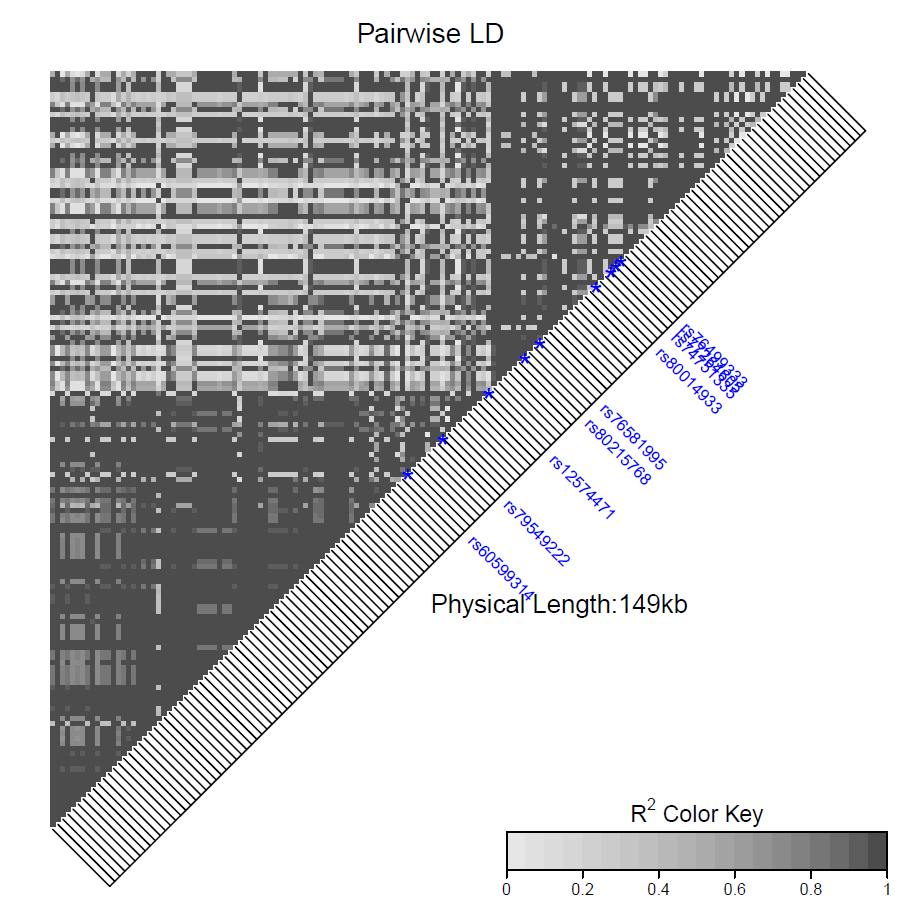

Supplement: Figure S13 [file peerj-03-1149-s013.png]

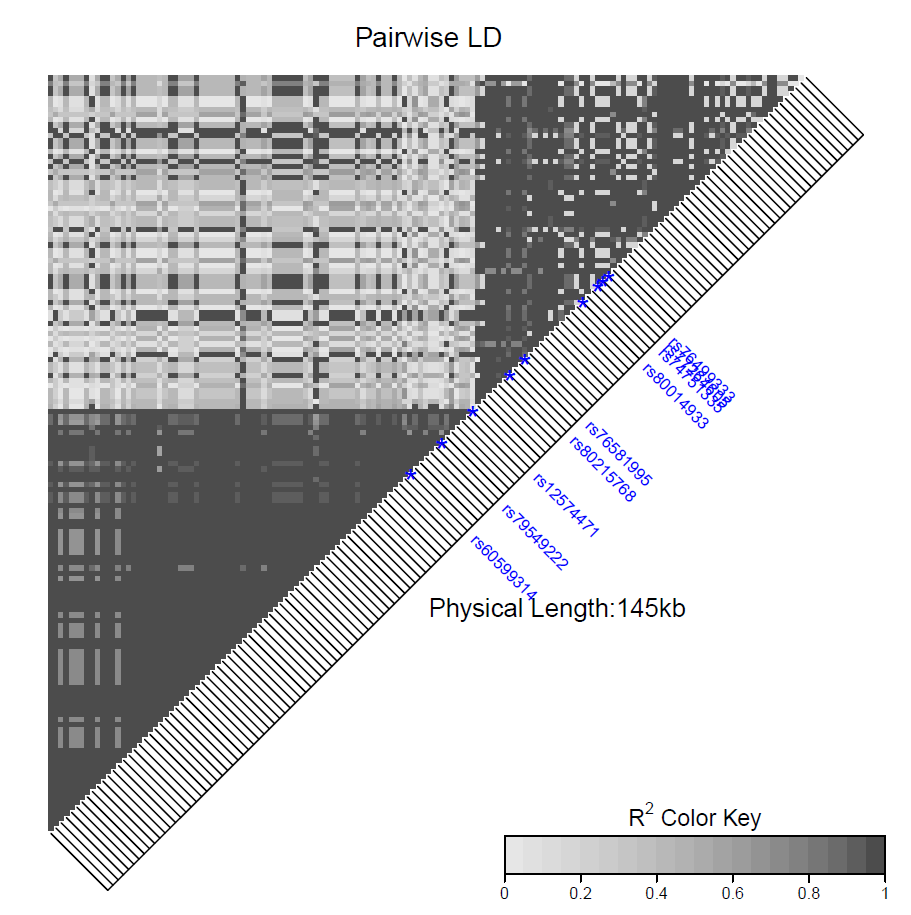

Supplement: Figure S14 [file peerj-03-1149-s014.png]

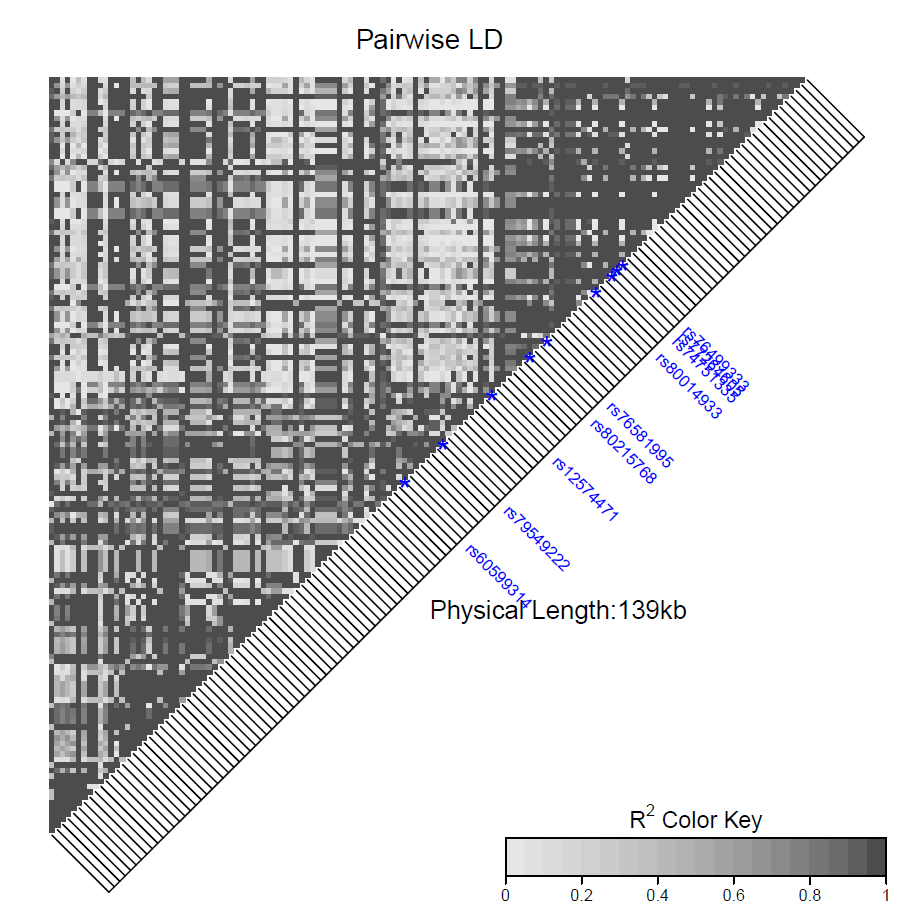

Supplement: Figure S15 [file peerj-03-1149-s015.png]

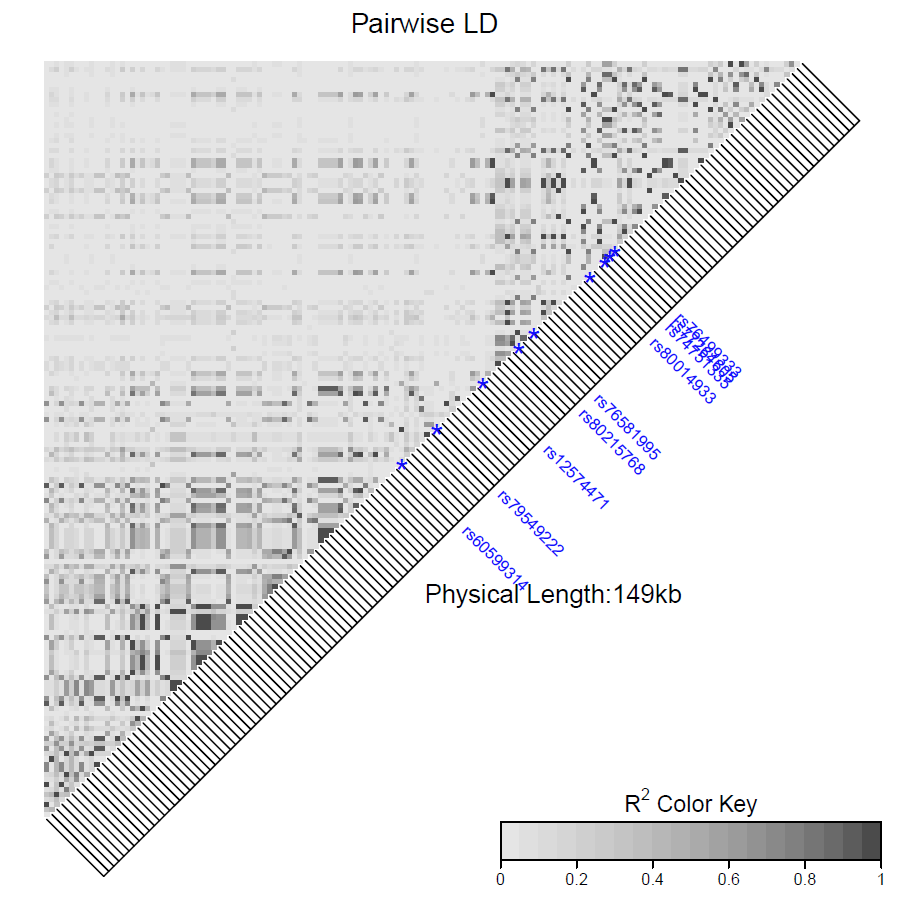

Supplement: Figure S16 [file peerj-03-1149-s016.png]

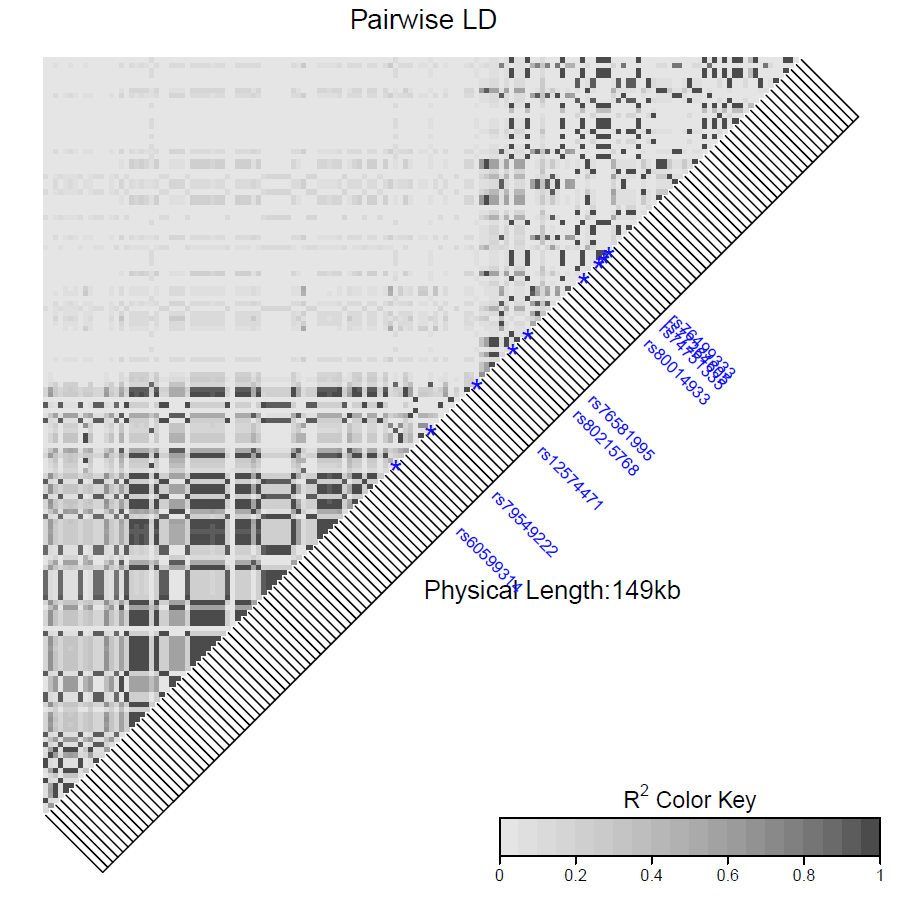

Supplement: Figure S17 [file peerj-03-1149-s017.png]

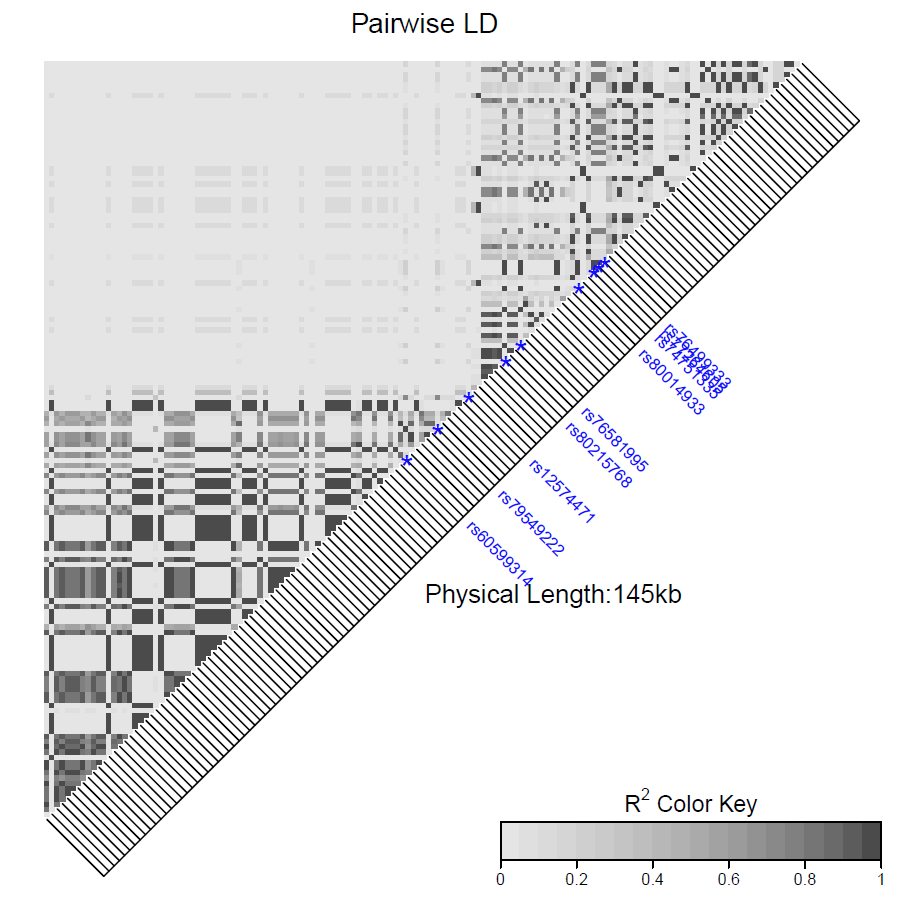

Supplement: Figure S18 [file peerj-03-1149-s018.png]

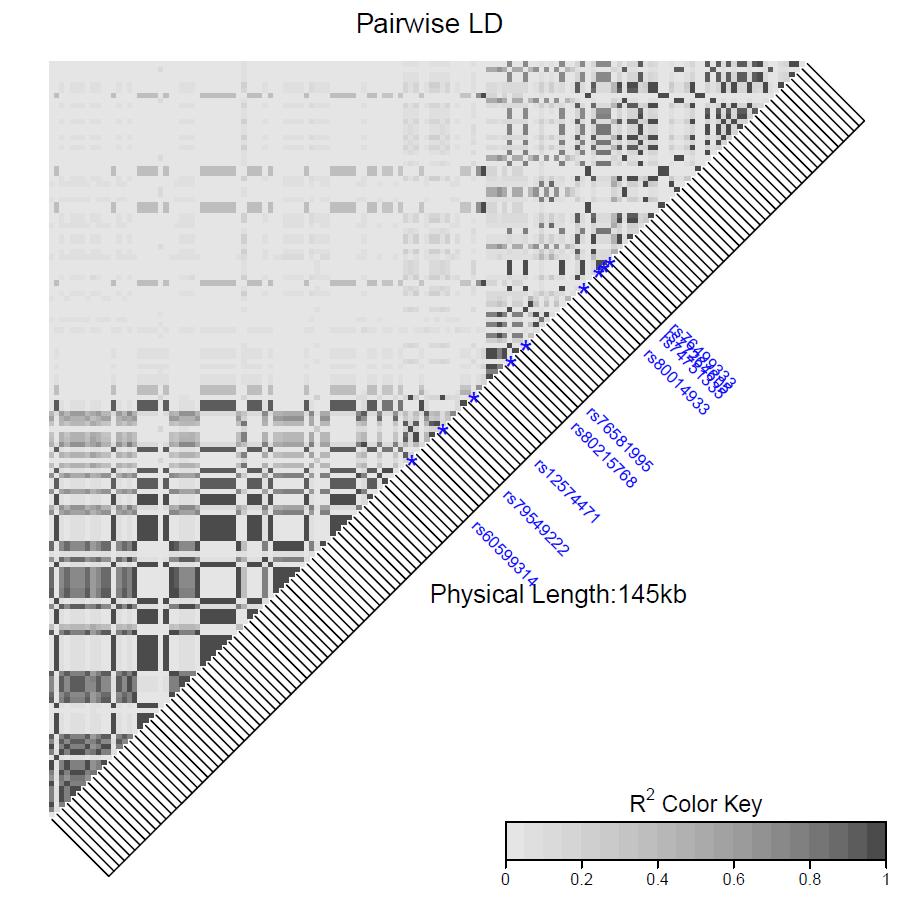

Supplement: Figure S19 [file peerj-03-1149-s019.png]

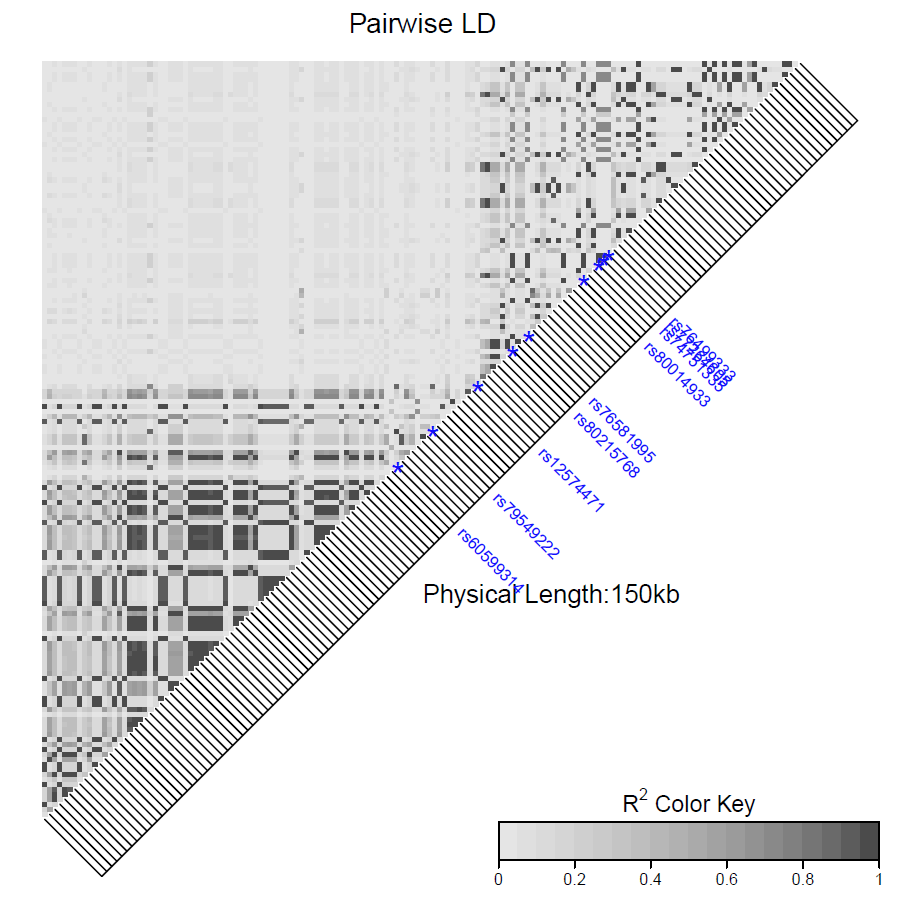

Supplement: Figure S20 [file peerj-03-1149-s020.png]

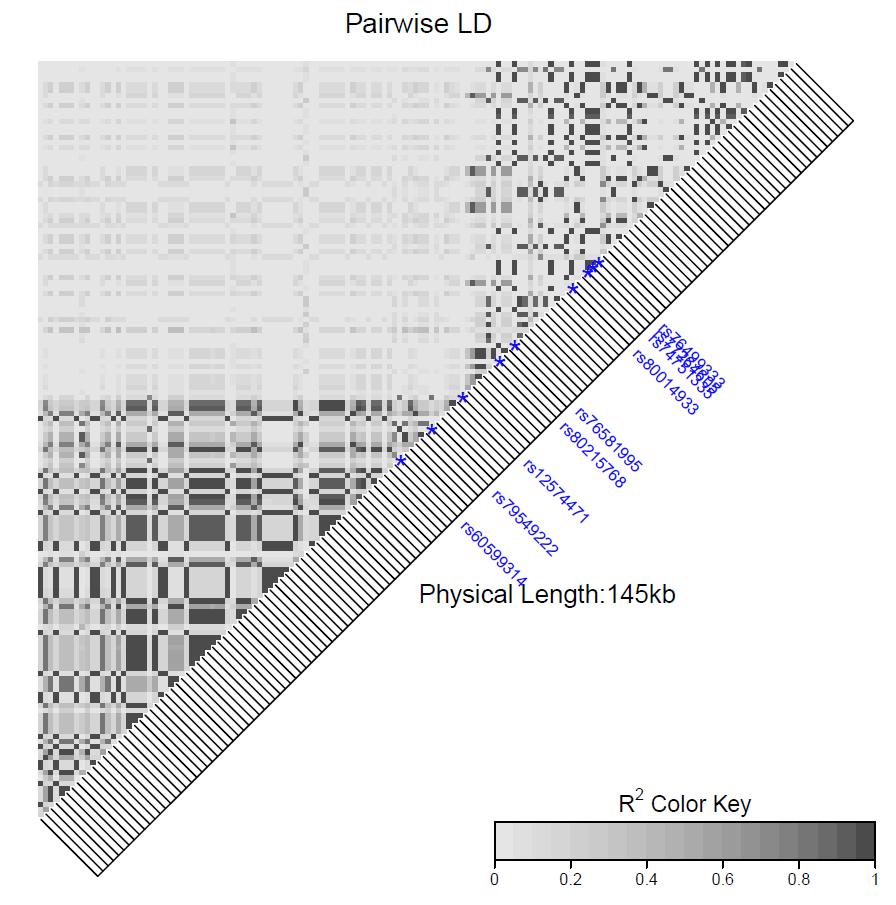

Supplement: Figure S21 [file peerj-03-1149-s021.png]

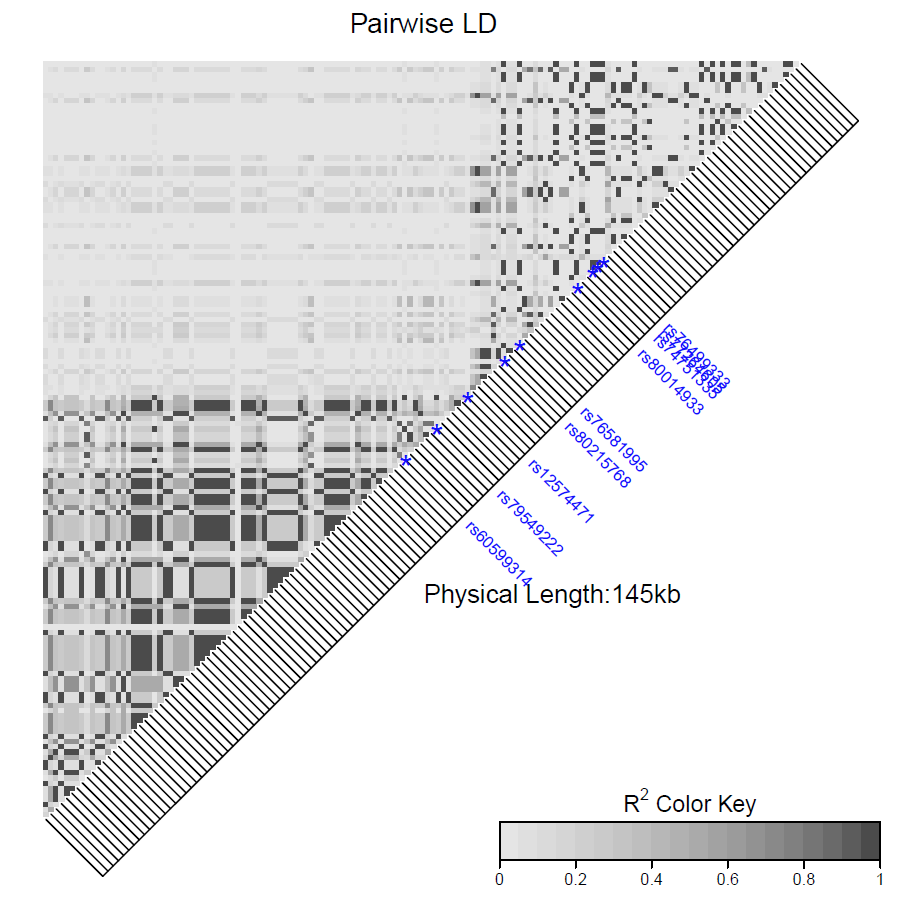

Supplement: Figure S22 [file peerj-03-1149-s022.png]

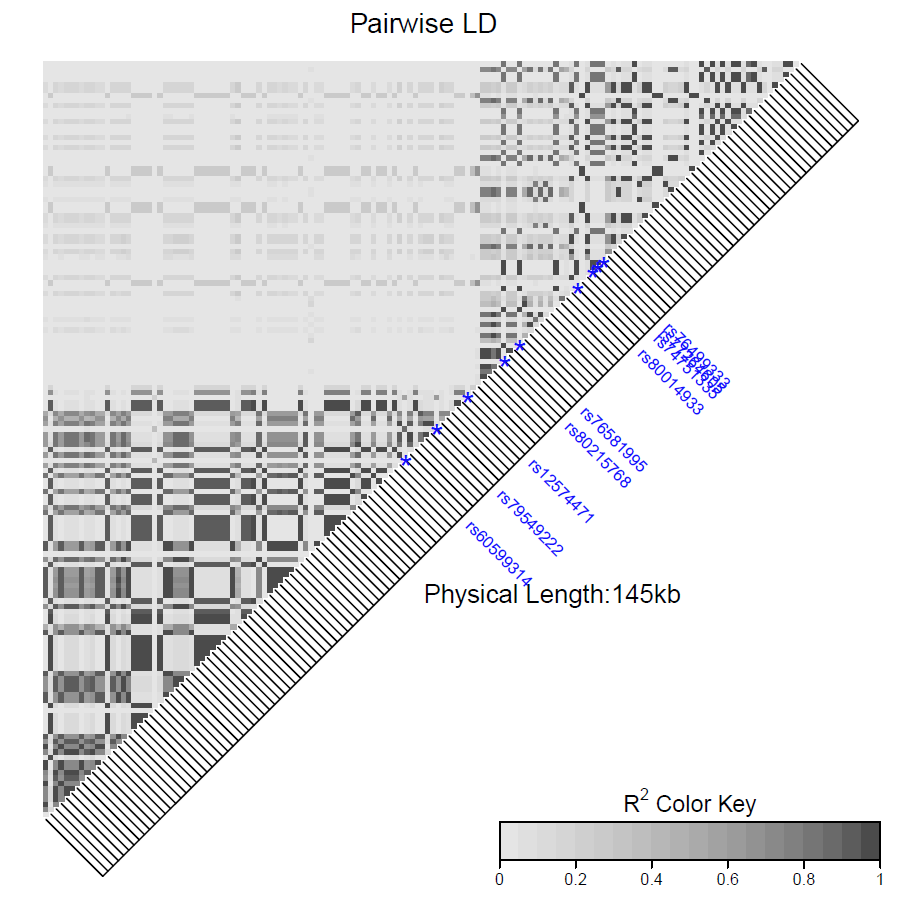

Supplement: Figure S23 [file peerj-03-1149-s023.png]

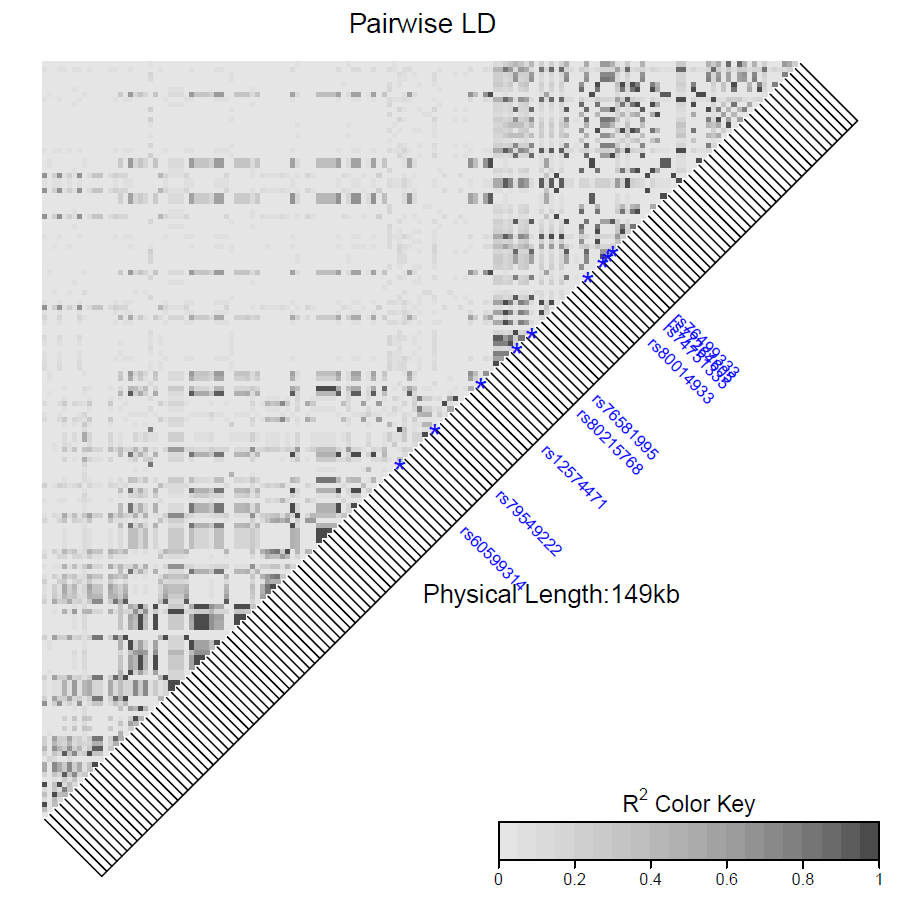

Supplement: Figure S24 [file peerj-03-1149-s024.png]

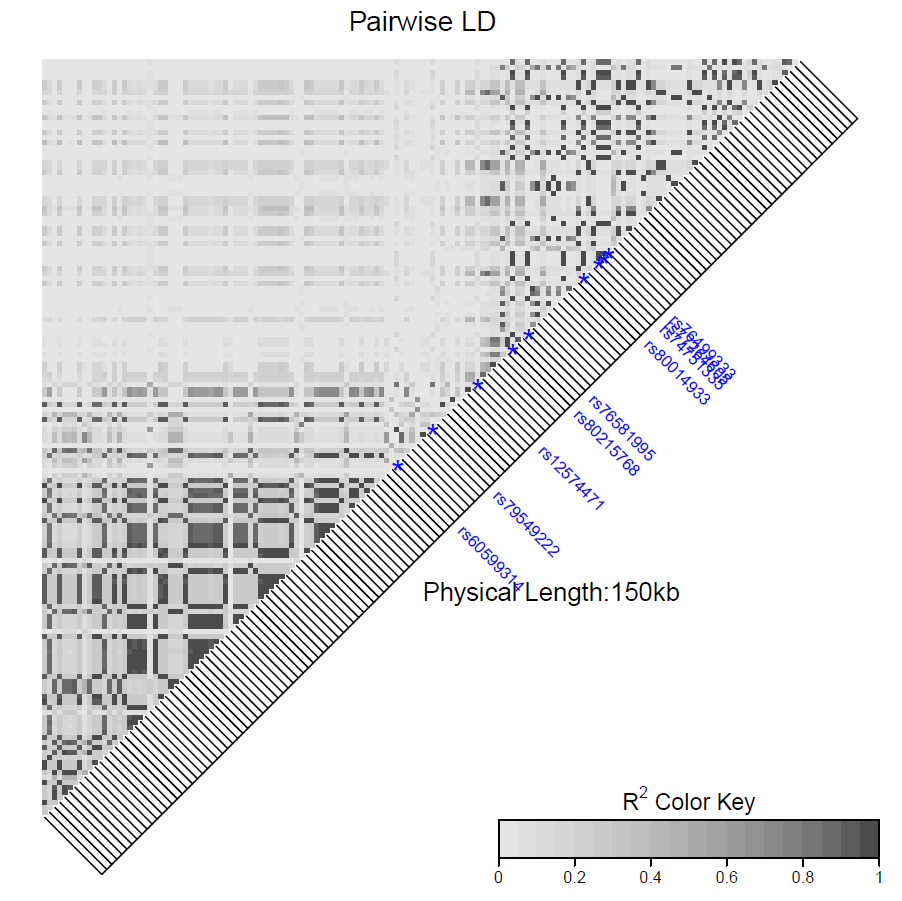

Supplement: Figure S25 [file peerj-03-1149-s025.png]

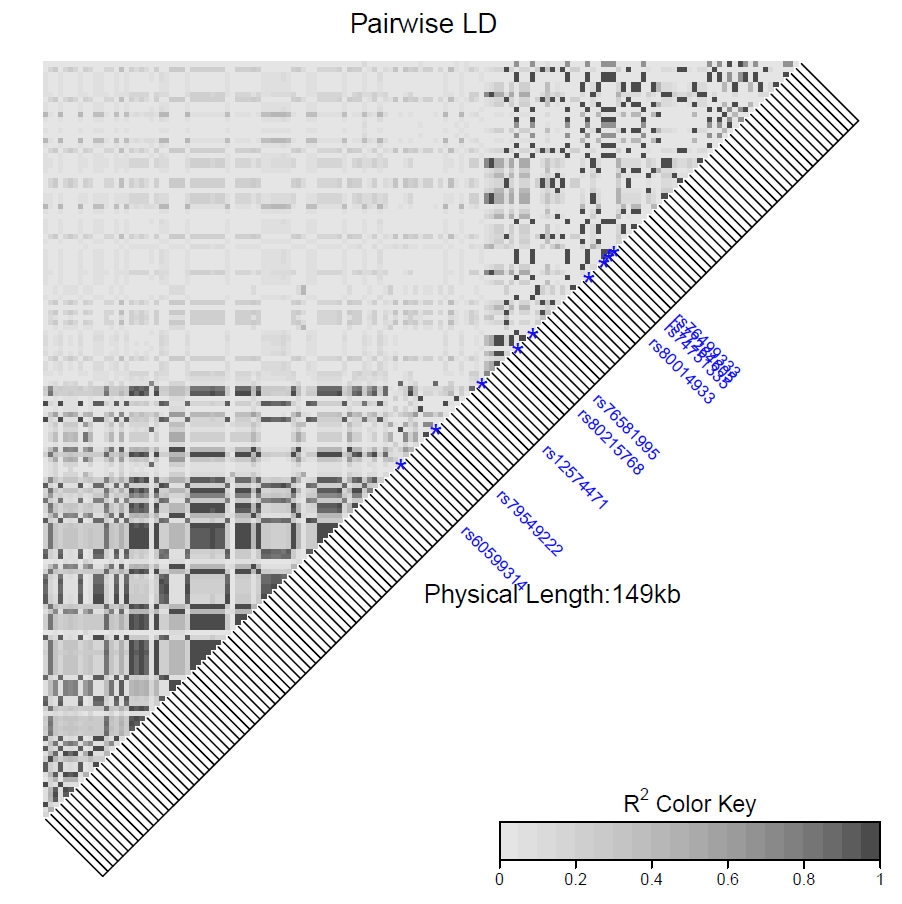

Supplement: Figure S26 [file peerj-03-1149-s026.png]

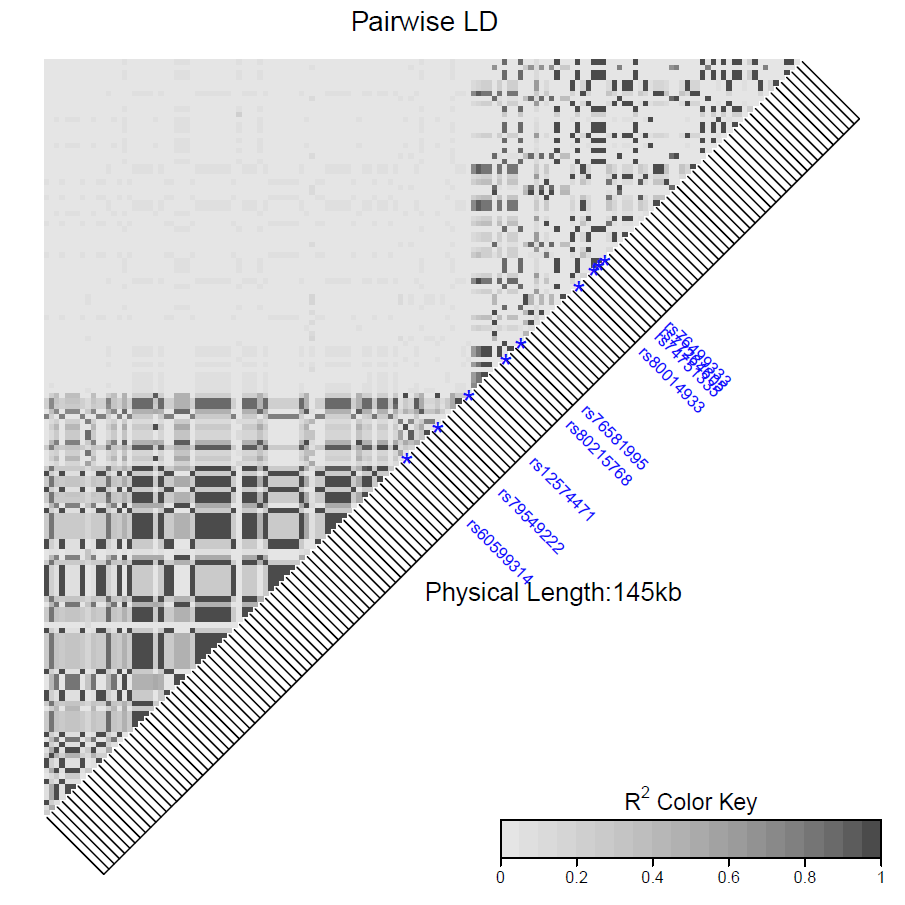

Supplement: Figure S27 [file peerj-03-1149-s027.png]

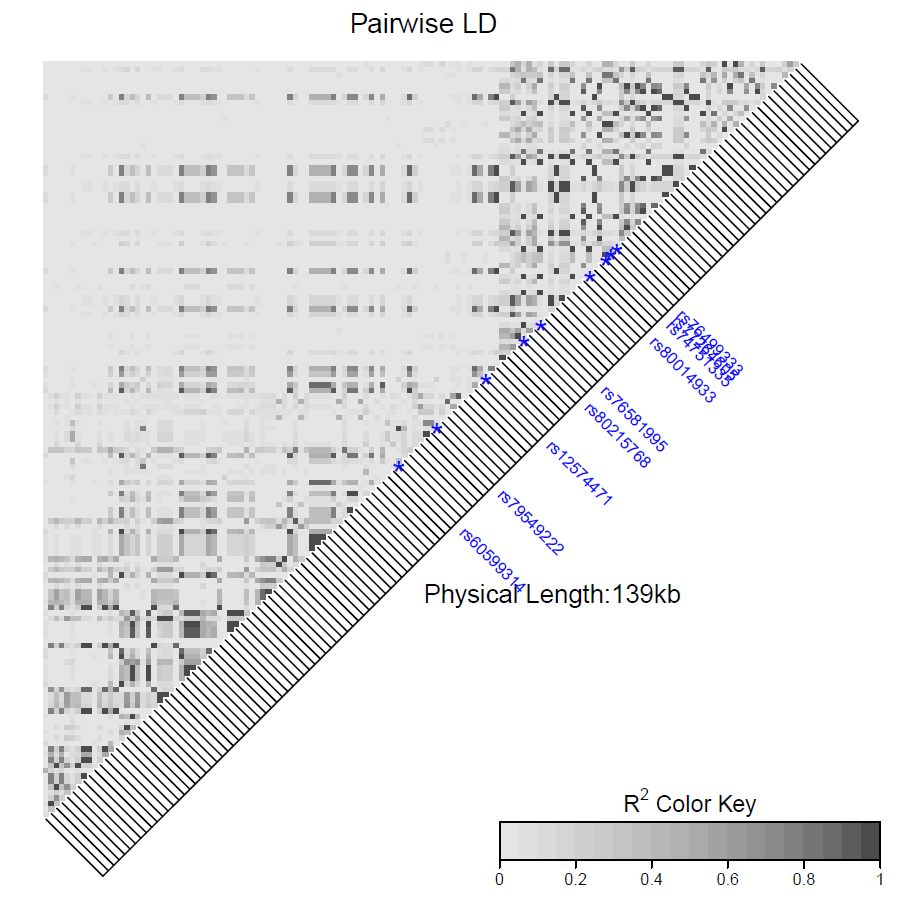

Supplement: Figure S28 [file peerj-03-1149-s028.png]
