# Supplementary material for: Selection in the dopamine receptor 2 gene: a candidate SNP study
Source: PeerJ. 2015 Aug 11;3:e1149. doi: 10.7717/peerj.1149 (PMC4540012; doi:10.7717/peerj.1149)
Supplement: Table S1 — These SNPs were excluded from the LD calculations. The population IBS was excluded entirely because 30 SNPs were flawed (which is nearly 20% of all SNPs). [file peerj-03-1149-s029.docx]

| Africa | ASW | rs75924850 |
| --- | --- | --- |
|  | LWK | rs75924850 |
|  | YRI | rs2242591, rs6278, rs1124491, rs2075654, rs75349786, rs72999670, rs77541954, rs4322431, rs72999677, rs11214614, rs75924850 |
| Europe | CEU | rs7126289 |
|  | FIN | rs7126289, rs7934416, rs4245145, rs4648319, rs4379875 |
|  | GBR | rs7126289, rs7934416, rs4245145, rs4648319, rs4379875 |
|  | IBS | rs2075652, rs7126289, rs7934416, rs4245145, rs4648319, rs4379875, rs4379875, rs80215768, rs76581995, chr11:113325866, rs80014933, rs74751335, rs77264605, rs76499333, rs74355206, rs75824731, rs10891553, rs77655590, rs12421616, rs73557296, rs4245149, rs60253148, rs7102650, rs55887984, rs75924850, rs117720003, rs79872790, rs79092434, rs7116768, rs1799978 |
|  | TSI | rs7126289, rs7934416, rs4245145, rs4648319, rs4379875 |
| East Asia | CHB | rs7126289, rs7934416, rs4245145, rs4648319, rs4379875 |
|  | CHS | rs7126289, rs7934416, rs4245145, rs4648319, rs4379875 |
|  | JPT | rs7126289, rs7934416, rs4245145, rs4648319, rs4379875 |
| America | CLM | none |
|  | MXL | none |
|  | PUR | rs75924850 |
