# Supplementary material for: Selection in the dopamine receptor 2 gene: a candidate SNP study
Source: PeerJ. 2015 Aug 11;3:e1149. doi: 10.7717/peerj.1149 (PMC4540012; doi:10.7717/peerj.1149)
Supplement: Table S2 — P, P (Simulation FST < sampleFST); He, expected heterozygosity; FST, Fixation Indices subpopulation to total population. P, posterior probability; log10 (PO), logarithm (base 10) of the posterior odds; q-value, false discovery rate (FDR) analogue of the P value; αi = locus-specific component (negative alpha suggests balancing selection, while positive alpha suggests directional selection); FST, Fixation Indices subpopulation to total population. ∗—the value of 1,000 means infinity (see BayeScan manual). [file peerj-03-1149-s030.docx]

| **Locus** | **P** | **H_E_** | **F_ST_** | **P** | **log10(PO)** | **q-value** | **α_i_** | **F_ST_** |
| --- | --- | --- | --- | --- | --- | --- | --- | --- |
| rs2242591 | 0.3390 | 0.3045 | 0.1200 | 0.0454 | -1.3227 | 0.5726 | -0.0101 | 0.1283 |
| rs11214603 | 0.9743 | 0.4330 | 0.2798 | **0.9964** | **2.4420** | 0.0015 | 0.9561 | 0.2485 |
| rs2734842 | 0.6401 | 0.5069 | 0.1851 | 0.0556 | -1.2300 | 0.5206 | 0.0205 | 0.1314 |
| rs6278 | 0.3390 | 0.3045 | 0.1200 | 0.0454 | -1.3227 | 0.5726 | -0.0098 | 0.1283 |
| rs6279 | 0.1893 | 0.4997 | 0.1049 | 0.0396 | -1.3847 | 0.6041 | -0.0046 | 0.1288 |
| rs6276 | 0.6401 | 0.5069 | 0.1851 | 0.0610 | -1.1872 | 0.5003 | 0.0197 | 0.1313 |
| rs2734841 | 0.0767 | 0.4956 | 0.0826 | 0.0568 | -1.2202 | 0.5167 | -0.0175 | 0.1277 |
| rs1124491 | 0.3390 | 0.3045 | 0.1200 | 0.0428 | -1.3495 | 0.5817 | -0.0098 | 0.1283 |
| rs1124492 | 0.3826 | 0.4137 | 0.1338 | 0.0410 | -1.3689 | 0.5932 | -0.0001 | 0.1292 |
| rs1124493 | 0.2013 | 0.4999 | 0.1068 | 0.0514 | -1.2660 | 0.5393 | -0.0062 | 0.1287 |
| rs1079595 | 0.1418 | 0.3201 | 0.0948 | 0.1202 | -0.8644 | 0.3432 | -0.0753 | 0.1233 |
| rs1079594 | 0.1418 | 0.3201 | 0.0948 | 0.1238 | -0.8498 | 0.3285 | -0.0793 | 0.1230 |
| rs6277 | 0.9770 | 0.4381 | 0.2829 | 0.9622 | 1.4057 | 0.0112 | 0.8430 | 0.2329 |
| rs6275 | 0.2530 | 0.4992 | 0.1145 | 0.0384 | -1.3986 | 0.6193 | -0.0033 | 0.1289 |
| rs1076560 | 0.1342 | 0.3201 | 0.0934 | 0.1282 | -0.8324 | 0.3209 | -0.0843 | 0.1226 |
| rs2511521 | 0.8009 | 0.5017 | 0.2018 | 0.0524 | -1.2572 | 0.5320 | 0.0177 | 0.1311 |
| rs2283265 | 0.1820 | 0.3216 | 0.1016 | 0.0970 | -0.9688 | 0.4444 | -0.0592 | 0.1245 |
| rs12363125 | 0.9853 | 0.4678 | 0.2997 | 0.9238 | 1.0835 | 0.0209 | 0.7663 | 0.2226 |
| rs2734839 | 0.8818 | 0.4902 | 0.2234 | 0.1060 | -0.9259 | 0.4182 | 0.0580 | 0.1359 |
| rs2734838 | 0.9683 | 0.4745 | 0.2695 | 0.5099 | 0.0172 | 0.1281 | 0.3776 | 0.1739 |
| rs2734837 | 0.8818 | 0.4902 | 0.2234 | 0.1062 | -0.9250 | 0.4127 | 0.0584 | 0.1359 |
| rs2440390 | **< 0.0001** | 0.1772 | 0.0188 | 0.9800 | 1.6901 | 0.0038 | -1.3782 | 0.0439 |
| rs1107162 | 0.9681 | 0.4742 | 0.2692 | 0.5489 | 0.0852 | 0.0806 | 0.4156 | 0.1787 |
| rs2075654 | 0.3957 | 0.3048 | 0.1287 | 0.0462 | -1.3147 | 0.5664 | -0.0076 | 0.1285 |
| rs1079727 | 0.1969 | 0.3213 | 0.1040 | 0.1162 | -0.8811 | 0.3769 | -0.0679 | 0.1238 |
| rs2002453 | 0.1608 | 0.4816 | 0.1060 | 0.0514 | -1.2660 | 0.5393 | -0.0142 | 0.1280 |
| rs2005313 | 0.1520 | 0.4812 | 0.1045 | 0.0476 | -1.3011 | 0.5599 | -0.0151 | 0.1279 |
| rs2245805 | 0.1551 | 0.4814 | 0.1051 | 0.0496 | -1.2823 | 0.5499 | -0.0136 | 0.1280 |
| rs2734836 | 0.1792 | 0.3213 | 0.1012 | 0.1108 | -0.9044 | 0.4013 | -0.0648 | 0.1240 |
| rs2734835 | 0.9859 | 0.4684 | 0.3011 | 0.9306 | 1.1273 | 0.0176 | 0.7844 | 0.2250 |
| rs1800498 | 0.9696 | 0.4751 | 0.2707 | 0.5397 | 0.0691 | 0.0974 | 0.4032 | 0.1770 |
| rs2234690 | 0.9696 | 0.4751 | 0.2707 | 0.5311 | 0.0541 | 0.1131 | 0.4008 | 0.1768 |
| rs2587548 | 0.9696 | 0.4751 | 0.2707 | 0.5357 | 0.0621 | 0.1053 | 0.4010 | 0.1767 |
| rs2734833 | 0.9347 | 0.4814 | 0.2451 | 0.2028 | -0.5944 | 0.2554 | 0.1362 | 0.1451 |
| rs67800399 | 0.9859 | 0.4684 | 0.3011 | 0.9274 | 1.1062 | 0.0193 | 0.7787 | 0.2243 |
| rs2734831 | 0.9848 | 0.4688 | 0.2987 | 0.9204 | 1.0630 | 0.0226 | 0.7696 | 0.2231 |
| rs1962262 | 0.1820 | 0.3216 | 0.1016 | 0.1008 | -0.9503 | 0.4237 | -0.0607 | 0.1243 |
| rs2075652 | 0.8792 | 0.2128 | 0.2432 | 0.0444 | -1.3328 | 0.5757 | 0.0106 | 0.1304 |
| rs11608185 | 0.9646 | 0.4754 | 0.2658 | 0.4599 | -0.0698 | 0.1512 | 0.3344 | 0.1687 |
| rs12808482 | 0.9646 | 0.4754 | 0.2658 | 0.4645 | -0.0618 | 0.1436 | 0.3382 | 0.1691 |
| rs1076563 | 0.9686 | 0.4745 | 0.2697 | 0.5433 | 0.0754 | 0.0891 | 0.4005 | 0.1766 |
| rs1076562 | 0.1607 | 0.4811 | 0.1060 | 0.0528 | -1.2537 | 0.5283 | -0.0157 | 0.1278 |
| rs1116313 | 0.9659 | 0.4752 | 0.2670 | 0.4901 | -0.0172 | 0.1357 | 0.3628 | 0.1721 |
| rs1079598 | 0.1732 | 0.3227 | 0.1002 | 0.1086 | -0.9142 | 0.4070 | -0.0621 | 0.1243 |
| rs1079597 | 0.0849 | 0.3402 | 0.0831 | 0.1176 | -0.8752 | 0.3572 | -0.0750 | 0.1233 |
| rs1079596 | 0.0774 | 0.3459 | 0.0812 | 0.1174 | -0.8760 | 0.3639 | -0.0684 | 0.1238 |
| rs1125394 | 0.0695 | 0.3454 | 0.0792 | 0.1206 | -0.8627 | 0.3360 | -0.0755 | 0.1233 |
| rs1125393 | 0.2480 | 0.3175 | 0.1065 | 0.1114 | -0.9017 | 0.3954 | -0.0693 | 0.1237 |
| rs2471857 | 0.0895 | 0.3401 | 0.0842 | 0.1184 | -0.8718 | 0.3503 | -0.0764 | 0.1233 |
| rs12798900 | 0.9861 | 0.4656 | 0.3015 | 0.9450 | 1.2350 | 0.0159 | 0.8104 | 0.2285 |
| rs2471856 | 0.4382 | 0.4919 | 0.1404 | 0.0388 | -1.3939 | 0.6118 | -0.0013 | 0.1291 |
| rs75349786 | **< 0.0001** | 0.0982 | 0.0112 | 0.6817 | 0.3308 | 0.0323 | -0.8099 | 0.0753 |
| rs2471855 | 0.2213 | 0.3194 | 0.1077 | 0.0996 | -0.9561 | 0.4394 | -0.0541 | 0.1249 |
| rs2471854 | 0.0853 | 0.3412 | 0.0832 | 0.1146 | -0.8879 | 0.3832 | -0.0704 | 0.1237 |
| rs7131627 | 0.9705 | 0.4730 | 0.2717 | 0.6153 | 0.2040 | 0.0717 | 0.4673 | 0.1849 |
| rs7131440 | 0.9714 | 0.4724 | 0.2728 | 0.6337 | 0.2381 | 0.0563 | 0.4826 | 0.1868 |
| rs7131465 | 0.9705 | 0.4718 | 0.2717 | 0.6555 | 0.2794 | 0.0405 | 0.5037 | 0.1894 |
| rs7131681 | 0.9714 | 0.4724 | 0.2728 | 0.6535 | 0.2756 | 0.0484 | 0.4915 | 0.1878 |
| rs11214605 | 0.9870 | 0.4647 | 0.3037 | 0.9638 | 1.4252 | 0.0102 | 0.8217 | 0.2299 |
| rs2471851 | 0.0904 | 0.3152 | 0.0809 | 0.1546 | -0.7378 | 0.2970 | -0.1121 | 0.1206 |
| rs2471850 | 0.4891 | 0.4953 | 0.1481 | 0.0374 | -1.4105 | 0.6264 | 0.0025 | 0.1295 |
| rs12800853 | 0.9881 | 0.4644 | 0.3066 | 0.9678 | 1.4778 | 0.0092 | 0.8377 | 0.2321 |
| rs11608109 | 0.9890 | 0.4664 | 0.3091 | 0.9702 | 1.5126 | 0.0082 | 0.8465 | 0.2334 |
| rs7103679 | 0.0949 | 0.3101 | 0.0818 | 0.1666 | -0.6991 | 0.2808 | -0.1203 | 0.1200 |
| rs4938017 | 0.9143 | 0.4877 | 0.2353 | 0.1964 | -0.6118 | 0.2639 | 0.1314 | 0.1445 |
| rs7122246 | 0.9889 | 0.4668 | 0.3088 | 0.9750 | 1.5910 | 0.0064 | 0.8481 | 0.2335 |
| rs7350522 | 0.2318 | 0.2945 | 0.1041 | 0.0766 | -1.0811 | 0.4641 | -0.0360 | 0.1262 |
| rs12364051 | 0.9716 | 0.4760 | 0.2729 | 0.6239 | 0.2199 | 0.0641 | 0.4752 | 0.1859 |
| rs78022226 | 0.5596 | 0.2914 | 0.1583 | 0.0588 | -1.2042 | 0.5086 | -0.0236 | 0.1272 |
| rs7126289 | 0.6176 | 0.0935 | 0.1444 | 0.3077 | -0.3523 | 0.1834 | 0.3613 | 0.1764 |
| ***rs60599314*** | **< 0.0001** | 0.2162 | 0.0110 | **0.9996** | **3.3977** | 0.0002 | -1.9167 | 0.0272 |
| rs4436578 | 0.6978 | 0.4447 | 0.1865 | 0.0498 | -1.2805 | 0.5429 | 0.0160 | 0.1309 |
| rs4587762 | 0.9876 | 0.4665 | 0.3051 | 0.9552 | 1.3287 | 0.0135 | 0.8214 | 0.2300 |
| rs4586205 | 0.5186 | 0.4971 | 0.1528 | 0.0368 | -1.4178 | 0.6287 | 0.0039 | 0.1296 |
| rs7934416 | 0.7497 | 0.1104 | 0.1896 | 0.0802 | -1.0594 | 0.4544 | 0.0438 | 0.1347 |
| rs17115583 | 0.1594 | 0.3726 | 0.0976 | 0.0544 | -1.2400 | 0.5245 | -0.0153 | 0.1279 |
| rs4620755 | 0.1554 | 0.3722 | 0.0969 | 0.0496 | -1.2823 | 0.5499 | -0.0172 | 0.1278 |
| ***rs79549222*** | **< 0.0001** | 0.2174 | 0.0106 | **1.0000** | **1000 *** | < 0.0001 | -1.9641 | 0.0260 |
| rs7125415 | 0.3607 | 0.3169 | 0.1233 | 0.0776 | -1.0750 | 0.4593 | -0.0384 | 0.1260 |
| rs11214607 | 0.0942 | 0.3028 | 0.0816 | 0.1296 | -0.8270 | 0.3132 | -0.0904 | 0.1222 |
| rs4648318 | 0.1803 | 0.4760 | 0.1090 | 0.0650 | -1.1578 | 0.4829 | -0.0231 | 0.1272 |
| rs4245145 | 0.8714 | 0.1319 | 0.2412 | 0.1006 | -0.9513 | 0.4290 | 0.0678 | 0.1376 |
| rs4648319 | 0.0907 | 0.2995 | 0.0810 | 0.1306 | -0.8232 | 0.3052 | -0.0865 | 0.1225 |
| rs4379875 | 0.8766 | 0.1337 | 0.2443 | 0.1004 | -0.9522 | 0.4342 | 0.0704 | 0.1379 |
| rs11214608 | **0.9903** | 0.4723 | 0.3068 | 0.9146 | 1.0297 | 0.0244 | 0.7472 | 0.2200 |
| rs76208665 | 0.9798 | 0.4812 | 0.2840 | 0.5281 | 0.0489 | 0.1206 | 0.3886 | 0.1752 |
| ***rs12574471*** | **< 0.0001** | 0.1873 | 0.0172 | **0.9962** | **2.4185** | 0.0017 | -1.5819 | 0.0364 |
| rs17529477 | 0.3477 | 0.3433 | 0.1259 | 0.0392 | -1.3893 | 0.6093 | 0.0076 | 0.1300 |
| rs17601612 | 0.6175 | 0.3687 | 0.1676 | 0.0642 | -1.1636 | 0.4873 | 0.0243 | 0.1319 |
| rs4245146 | 0.4248 | 0.5044 | 0.1384 | 0.0356 | -1.4327 | 0.6333 | 0.0011 | 0.1293 |
| rs4245147 | 0.4248 | 0.5044 | 0.1384 | 0.0406 | -1.3734 | 0.5960 | -0.0005 | 0.1292 |
| rs4936270 | 0.8616 | 0.3693 | 0.2207 | 0.0378 | -1.4057 | 0.6241 | 0.0058 | 0.1298 |
| rs4936271 | 0.2498 | 0.5028 | 0.1140 | 0.0422 | -1.3559 | 0.5846 | -0.0064 | 0.1286 |
| ***rs80215768*** | **0.0099** | 0.1249 | 0.0304 | **0.9986** | **2.8532** | 0.0008 | -1.7050 | 0.0328 |
| rs4936272 | 0.2515 | 0.5027 | 0.1143 | 0.0438 | -1.3390 | 0.5787 | -0.0055 | 0.1287 |
| rs4274224 | 0.3622 | 0.5042 | 0.1297 | 0.0396 | -1.3847 | 0.6041 | -0.0003 | 0.1292 |
| ***rs76581995*** | **0.0099** | 0.1249 | 0.0304 | **0.9988** | **2.9202** | 0.0006 | -1.7062 | 0.0328 |
| rs77195172 | **0.0008** | 0.1186 | 0.0162 | 0.9772 | 1.6320 | 0.0055 | -1.4064 | 0.0430 |
| rs4245148 | 0.8362 | 0.3669 | 0.2130 | 0.0416 | -1.3624 | 0.5904 | 0.0056 | 0.1297 |
| rs55697087 | 0.9231 | 0.5045 | 0.2400 | 0.1166 | -0.8794 | 0.3705 | 0.0650 | 0.1367 |
| rs4460839 | 0.8432 | 0.3669 | 0.2150 | 0.0346 | -1.4455 | 0.6399 | 0.0067 | 0.1298 |
| rs72999670 | 0.0457 | 0.3450 | 0.0718 | 0.0468 | -1.3088 | 0.5632 | 0.0124 | 0.1305 |
| rs4581480 | 0.8514 | 0.3656 | 0.2175 | 0.0348 | -1.4429 | 0.6377 | 0.0059 | 0.1298 |
| chr11:113325866 | 0.0104 | 0.1251 | 0.0309 | **0.9984** | **2.7951** | 0.0009 | -1.6918 | 0.0331 |
| rs73557283 | **0.0014** | 0.1638 | 0.0144 | 0.9790 | 1.6685 | 0.0047 | -1.4261 | 0.0427 |
| rs77541954 | 0.0120 | 0.1316 | 0.0320 | 0.4525 | -0.0828 | 0.1658 | -0.4797 | 0.0958 |
| rs7122454 | 0.0424 | 0.3447 | 0.0706 | 0.1686 | -0.6929 | 0.2725 | -0.1322 | 0.1193 |
| ***rs80014933*** | **0.0099** | 0.1280 | 0.0304 | **0.9982** | **2.7439** | 0.0010 | -1.6935 | 0.0332 |
| rs7948028 | 0.5684 | 0.3598 | 0.1594 | 0.0640 | -1.1650 | 0.4917 | 0.0242 | 0.1319 |
| rs10891550 | 0.0272 | 0.3511 | 0.0640 | 0.2423 | -0.4953 | 0.2293 | -0.2168 | 0.1133 |
| ***rs74751335*** | **0.0072** | 0.1417 | 0.0266 | **0.9980** | **2.6980** | 0.0011 | -1.7267 | 0.0322 |
| ***rs77264605*** | **0.0072** | 0.1417 | 0.0266 | **0.9994** | **3.2215** | 0.0003 | -1.7108 | 0.0327 |
| ***rs76499333*** | **0.0092** | 0.1251 | 0.0299 | **0.9990** | **2.9995** | 0.0005 | -1.7042 | 0.0327 |
| rs7131056 | 0.0276 | 0.5005 | 0.0646 | 0.1132 | -0.8939 | 0.3894 | -0.0656 | 0.1240 |
| rs11214611 | 0.0289 | 0.3509 | 0.0648 | 0.2587 | -0.4573 | 0.2114 | -0.2297 | 0.1124 |
| rs4936274 | 0.8579 | 0.3584 | 0.2195 | 0.0420 | -1.3580 | 0.5875 | 0.0061 | 0.1298 |
| rs4648317 | 0.0285 | 0.3515 | 0.0646 | 0.2449 | -0.4891 | 0.2205 | -0.2139 | 0.1134 |
| rs10891551 | 0.0517 | 0.3446 | 0.0738 | 0.1662 | -0.7003 | 0.2890 | -0.1228 | 0.1199 |
| rs74355206 | 0.0340 | 0.1342 | 0.0424 | **0.9944** | **2.2493** | 0.0027 | -1.5322 | 0.0380 |
| rs4322431 | 0.0577 | 0.3545 | 0.0758 | 0.0588 | -1.2042 | 0.5086 | 0.0182 | 0.1312 |
| rs7117915 | 0.0440 | 0.3433 | 0.0712 | 0.2144 | -0.5639 | 0.2468 | -0.1743 | 0.1162 |
| rs10891552 | 0.5066 | 0.2641 | 0.1488 | 0.0394 | -1.3870 | 0.6067 | 0.0011 | 0.1293 |
| rs4337071 | 0.5780 | 0.3598 | 0.1610 | 0.0634 | -1.1694 | 0.4961 | 0.0268 | 0.1322 |
| rs4630328 | 0.5842 | 0.3592 | 0.1620 | 0.0662 | -1.1493 | 0.4783 | 0.0296 | 0.1325 |
| rs72999677 | 0.0469 | 0.3466 | 0.0722 | 0.0488 | -1.2898 | 0.5533 | 0.0118 | 0.1304 |
| rs11214612 | 0.8520 | 0.3568 | 0.2176 | 0.0360 | -1.4277 | 0.6310 | 0.0049 | 0.1297 |
| rs11214613 | 0.8485 | 0.3572 | 0.2166 | 0.0400 | -1.3801 | 0.5987 | 0.0061 | 0.1298 |
| rs11214614 | 0.0469 | 0.3466 | 0.0722 | 0.0484 | -1.2935 | 0.5566 | 0.0119 | 0.1305 |
| rs4350392 | 0.0415 | 0.3439 | 0.0703 | 0.2218 | -0.5450 | 0.2381 | -0.1893 | 0.1152 |
| rs11601054 | 0.5722 | 0.3602 | 0.1600 | 0.0686 | -1.1327 | 0.4736 | 0.0283 | 0.1324 |
| rs61902787 | 0.5722 | 0.3602 | 0.1600 | 0.0700 | -1.1233 | 0.4689 | 0.0298 | 0.1326 |
| rs75824731 | 0.0370 | 0.1355 | 0.0435 | **0.9956** | **2.3545** | 0.0021 | -1.5273 | 0.0381 |
| rs10891553 | 0.8331 | 0.3552 | 0.2121 | 0.0352 | -1.4378 | 0.6355 | 0.0037 | 0.1295 |
| rs77655590 | 0.0297 | 0.1397 | 0.0421 | **0.9958** | **2.3748** | 0.0019 | -1.5357 | 0.0379 |
| rs12421616 | 0.6103 | 0.2519 | 0.1690 | 0.0386 | -1.3962 | 0.6143 | 0.0002 | 0.1292 |
| rs73557296 | **0.0033** | 0.1752 | 0.0417 | 0.9538 | 1.3147 | 0.0146 | -1.2335 | 0.0498 |
| rs4245149 | 0.0214 | 0.3498 | 0.0607 | 0.3739 | -0.2239 | 0.1742 | -0.3629 | 0.1030 |
| rs60253148 | **0.0033** | 0.1752 | 0.0417 | 0.9552 | 1.3287 | 0.0135 | -1.2386 | 0.0496 |
| rs7102650 | 0.0182 | 0.1493 | 0.0360 | **0.9966** | **2.4670** | 0.0013 | -1.5757 | 0.0367 |
| rs55887984 | 0.5262 | 0.2616 | 0.1526 | 0.0378 | -1.4057 | 0.6241 | -0.0010 | 0.1291 |
| rs75924850 | 0.3384 | 0.0832 | 0.0874 | 0.0954 | -0.9768 | 0.4494 | -0.0538 | 0.1251 |
| rs117720003 | 0.0307 | 0.1342 | 0.0412 | **0.9952** | **2.3166** | 0.0023 | -1.5471 | 0.0375 |
| rs4938019 | 0.0363 | 0.3435 | 0.0682 | 0.2627 | -0.4483 | 0.2023 | -0.2321 | 0.1121 |
| rs10891554 | 0.8473 | 0.3567 | 0.2162 | 0.0384 | -1.3986 | 0.6193 | 0.0040 | 0.1296 |
| rs61902807 | 0.5694 | 0.3597 | 0.1595 | 0.0572 | -1.2169 | 0.5127 | 0.0258 | 0.1321 |
| rs10789943 | 0.0363 | 0.3435 | 0.0682 | 0.2775 | -0.4157 | 0.1929 | -0.2458 | 0.1110 |
| rs10789944 | 0.0188 | 0.3513 | 0.0591 | 0.4563 | -0.0761 | 0.1586 | -0.4585 | 0.0965 |
| rs79872790 | 0.0282 | 0.1390 | 0.0414 | **0.9932** | **2.1644** | 0.0029 | -1.4841 | 0.0397 |
| rs79092434 | 0.0147 | 0.1511 | 0.0336 | **0.9944** | **2.2493** | 0.0027 | -1.5862 | 0.0365 |
| rs7116768 | 0.8284 | 0.3655 | 0.2108 | 0.0320 | -1.4806 | 0.6421 | 0.0024 | 0.1294 |
| rs1799978 | 0.0204 | 0.1709 | 0.0374 | 0.9740 | 1.5735 | 0.0073 | -1.3142 | 0.0461 |
