# Supplementary material for: Selection in the dopamine receptor 2 gene: a candidate SNP study
Source: PeerJ. 2015 Aug 11;3:e1149. doi: 10.7717/peerj.1149 (PMC4540012; doi:10.7717/peerj.1149)
Supplement: Table S3A — Comparison of the candidate loci under selection found both samples; (a) shows the calculations of the MAF > 0.05 sample and the (b) the calculations of the MAF > 0.01 sample. P, P (Simulation FST < sample FST); He, expected heterozygosity; FST, Fixation Indices subpopulation to total population. P, posterior probability; log10 (PO), logarithm (base 10) of the posterior odds; q-value, false discovery rate (FDR) analogue of the P value; αi, locus-specific component (negative alpha suggests balancing selection, while positive alpha suggests directional selection); FST, Fixation Indices subpopulation to total population. [file peerj-03-1149-s031.docx]

| **> 0.05 MAF** |  | **LOSITAN** |  |  |  | **BayeScan** |  |  |
| --- | --- | --- | --- | --- | --- | --- | --- | --- |
| **Locus** | **P** | **H_E_** | **F_ST_** | **P** | **log10(PO)** | **q-value** | **α_i_** | **F_ST_** |
| ***rs60599314 (1)*** | **< 0.0001** | 0.2162 | 0.0110 | **0.9996** | **3.3977** | 0.0002 | -1.9167 | 0.0272 |
| ***rs79549222 (2)*** | **< 0.0001** | 0.2174 | 0.0106 | **1.0000** | **1000.0000** | < 0.0001 | -1.9641 | 0.0260 |
| ***rs12574471 (3)*** | **< 0.0001** | 0.1873 | 0.0172 | **0.9962** | **2.4185** | 0.0017 | -1.5819 | 0.0364 |
| ***rs80215768 (4)*** | **0.0099** | 0.1249 | 0.0304 | **0.9986** | **2.8532** | 0.0008 | -1.7050 | 0.0328 |
| ***rs76581995 (5)*** | **0.0099** | 0.1249 | 0.0304 | **0.9988** | **2.9202** | 0.0006 | -1.7062 | 0.0328 |
| ***rs80014933 (6)*** | **0.0099** | 0.1280 | 0.0304 | **0.9982** | **2.7439** | 0.0010 | -1.6935 | 0.0332 |
| ***rs74751335 (7)*** | **0.0072** | 0.1417 | 0.0266 | **0.9980** | **2.6980** | 0.0011 | -1.7267 | 0.0322 |
| ***rs77264605 (8)*** | **0.0072** | 0.1417 | 0.0266 | **0.9994** | **3.2215** | 0.0003 | -1.7108 | 0.0327 |
| ***rs76499333 (9)*** | **0.0092** | 0.1251 | 0.0299 | **0.9990** | **2.9995** | 0.0005 | -1.7042 | 0.0327 |
| rs6277 | 0.9770 | 0.4381 | 0.2829 | 0.9622 | 1.4057 | 0.0112 | 0.8430 | 0.2329 |
| rs12800853 | 0.9881 | 0.4644 | 0.3066 | 0.9678 | 1.4778 | 0.0092 | 0.8377 | 0.2321 |
| rs11608109 | 0.9890 | 0.4664 | 0.3091 | 0.9702 | 1.5126 | 0.0082 | 0.8465 | 0.2334 |
