# Supplementary material for: Selection in the dopamine receptor 2 gene: a candidate SNP study
Source: PeerJ. 2015 Aug 11;3:e1149. doi: 10.7717/peerj.1149 (PMC4540012; doi:10.7717/peerj.1149)
Supplement: Table S3B — Comparison of the candidate loci under selection found both samples; (a) shows the calculations of the MAF > 0.05 sample and the (b) the calculations of the MAF > 0.01 sample. P P (Simulation FST < sample FST); He, expected heterozygosity; FST, Fixation Indices subpopulation to total population. P, posterior probability; log10 (PO), logarithm (base 10) of the posterior odds; q-value, false discovery rate (FDR) analogue of the P value; αi, locus-specific component (negative alpha suggests balancing selection, while positive alpha suggests directional selection); FST, Fixation Indices subpopulation to total population. [file peerj-03-1149-s032.docx]

| **> 0.01 MAF** |  | **LOSITAN** |  |  |  | **BayeScan** |  |  |
| --- | --- | --- | --- | --- | --- | --- | --- | --- |
| **Locus** | **P** | **H_E_** | **F_ST_** | **P** | **log10(PO)** | **q-value** | **α_i_** | **F_ST_** |
| ***rs60599314 (1)*** | **< 0.0001** | 0.2162 | 0.0110 | **0.9994** | **3.2215** | 0.0004 | -1.8805 | 0.0251 |
| ***rs79549222 (2)*** | **< 0.0001** | 0.2174 | 0.0106 | **0.9998** | **3.6988** | 0.0002 | -1.9220 | 0.0243 |
| rs12574471 (3) | **0.0002** | 0.1873 | 0.0172 | 0.9878 | 1.9082 | 0.0064 | -1.4660 | 0.0365 |
| rs80215768 (4) | 0.0187 | 0.1249 | 0.0304 | **0.9958** | **2.3748** | 0.0015 | -1.6190 | 0.0320 |
| rs76581995 (5) | 0.0187 | 0.1249 | 0.0304 | **0.9946** | **2.2652** | 0.0031 | -1.6179 | 0.0321 |
| rs80014933 (6) | 0.0187 | 0.1280 | 0.0304 | **0.9934** | **2.1775** | 0.0040 | -1.5901 | 0.0327 |
| rs74751335 (7) | 0.0114 | 0.1417 | 0.0266 | **0.9944** | **2.2493** | 0.0034 | -1.6147 | 0.0322 |
| rs77264605 (8) | 0.0114 | 0.1417 | 0.0266 | **0.9948** | **2.2816** | 0.0027 | -1.6072 | 0.0324 |
| rs76499333 (9) | 0.0177 | 0.1251 | 0.0299 | **0.9948** | **2.2816** | 0.0027 | -1.5970 | 0.0327 |
| ***rs6277*** | **0.9912** | 0.4381 | 0.2829 | **0.9912** | **2.0516** | 0.0050 | 0.9006 | 0.2353 |
| ***rs12800853*** | **0.9963** | 0.4644 | 0.3066 | **0.9916** | **2.0720** | 0.0047 | 0.8934 | 0.2341 |
| ***rs11608109*** | **0.9966** | 0.4664 | 0.3091 | **0.9916** | **2.0720** | 0.0047 | 0.9048 | 0.2359 |
